# Zeaxanthin is required for eyespot formation and phototaxis in *Euglena gracilis*

**DOI:** 10.1093/plphys/kiad001

**Published:** 2023-01-06

**Authors:** Shun Tamaki, Kazunari Ozasa, Toshihisa Nomura, Marumi Ishikawa, Koji Yamada, Kengo Suzuki, Keiichi Mochida

**Affiliations:** Microalgae Production Control Technology Laboratory, RIKEN Baton Zone Program, Yokohama 230-0045, Japan; RIKEN Center for Advanced Photonics, Wako, Saitama 351-0198, Japan; Microalgae Production Control Technology Laboratory, RIKEN Baton Zone Program, Yokohama 230-0045, Japan; RIKEN Center for Sustainable Resource Science, Yokohama 230-0045, Japan; Microalgae Production Control Technology Laboratory, RIKEN Baton Zone Program, Yokohama 230-0045, Japan; Microalgae Production Control Technology Laboratory, RIKEN Baton Zone Program, Yokohama 230-0045, Japan; euglena Co., Ltd., Tokyo 108-0014, Japan; Microalgae Production Control Technology Laboratory, RIKEN Baton Zone Program, Yokohama 230-0045, Japan; euglena Co., Ltd., Tokyo 108-0014, Japan; Microalgae Production Control Technology Laboratory, RIKEN Baton Zone Program, Yokohama 230-0045, Japan; RIKEN Center for Sustainable Resource Science, Yokohama 230-0045, Japan; Kihara Institute for Biological Research, Yokohama City University, Yokohma 244-0812, Japan; School of Information and Data Sciences, Nagasaki University, Nagasaki 852-8521, Japan

## Abstract

The eyespot apparatus is an organelle that forms carotenoid-rich globules in diverse flagellated microalgae and functions in phototaxis. The euglenophytes have structurally and functionally distinct eyespot apparatuses from chlorophytes. β-Carotene is the most abundant pigment detected in chlorophytes’ eyespots, while xanthophylls such as zeaxanthin and diadinoxanthin have been suggested to function in euglenophytes’ eyespots. Here, we investigated the association between carotenoid composition and eyespot formation via pathway-scale mutagenesis using clustered regularly interspaced short palindromic repeats (CRISPR)/CRISPR-associated protein 9 (Cas9)-mediated genome editing in the euglenophyte *Euglena gracilis*. *Lycopene cyclase* (*lcy*) mutants exhibited sole lycopene accumulation, defective red eyespots, and phototactic insensitivity. Conversely, β-carotene hydroxylase (*cytochrome P450 97h1, cyp97h1*) mutants accumulated β-carotene and its hydroxylated products β-cryptoxanthin and zeaxanthin and formed phototactic eyespot apparatuses, while *cyp97h1 cyp97f2* double mutants were deficient in β-carotene hydroxylation and mostly lacked functional eyespots. Thus, zeaxanthin is required for the stable formation of functional eyespots in *E. gracilis*, highlighting evolutionary differences between euglenophytes and chlorophytes in the metabolic regulation of photoreactive organelle formation.

## Teaser

Zeaxanthin is required for eyespot formation and phototaxis in *Euglena gracilis*, highlighting the evolutionary diversity of the photosensing mechanism between euglenophytes and chlorophytes.

## Introduction

Microalgae evolved phototactic responses that optimize photosynthesis in response to the light environment. These microalgae usually move toward (positive phototaxis) and away from (negative phototaxis) a light source (Häder et al., 1981; Häder and Iseki, 2017). Accurate phototaxis in a wide range of microalgae, including chlorophytes, euglenophytes, and dinoflagellates, requires the eyespot apparatus, an assembly of carotenoid-rich globules, which give eyespots their red color (Colley and Nilsson, 2016). Carotenoids play a central role in photosensing mechanisms, which independently evolved in phylogenetic groups of phototactic microalgae (Colley and Nilsson, 2016).

Carotenoids are isoprenoid compounds with a C_40_ backbone; they function as photosynthetic pigments and hydrophobic antioxidants in photosynthetic organisms including microalgal species (Huang et al., 2017; Jacob-Lopes et al., 2020; Tamaki et al., 2021a). Chlorophytes synthesize carotenoids in chloroplasts, and their eyespot globules are derived from β-carotene-rich plastoglobules (Pick et al., 2019). The eyespot globules in *Chlamydomonas reinhardtii* form two carotenoid-rich layers and localize inside the chloroplast close to the photoreceptor protein channel rhodopsin (Foster and Smyth, 1980). The *C. reinhardtii lts1-211* mutant, which lacks a red eyespot, shows opposite phototactic responses to those of the wild-type (WT) and has less carotenoids (Ueki et al., 2016). The causal mutation of the *lts1-211* mutant was identified in the gene encoding phytoene synthase, the rate-limiting enzyme in carotenoid biosynthesis (Ueki et al., 2016). *Euglena gracilis*, a unicellular flagellated euglenophyte, also synthesizes carotenoids, such as β-carotene, diadinoxanthin, diatoxanthin, and neoxanthin (Kato et al., 2017), and forms red eyespot apparatuses (Kivic and Vesk, 1972). In *E. gracilis*, RNA interference (RNAi)–based suppression of phytoene synthase (*EgcrtB*) resulted in reduced carotenoid accumulation in the eyespot apparatus and loss of phototaxis (Kato et al., 2020; Tamaki et al., 2020). Thus, carotenoid biosynthesis is important for eyespot pigmentation and essential for phototaxis across diverse phyla of microalgae.

Contrary to the situation in *C. reinhardtii*, eyespot formation in *E. gracilis* is independent of chloroplast development. Electron microscopy and photomovement analyses demonstrated that chloroplast-deficient *E. gracilis* strains, such as SM-ZK and some RNAi-based *EgcrtB*-suppression lines, form a functional eyespot despite their chloroplast development deficiency, marked reduction of total carotenoids, and altered carotenoid composition compared with the WT (Tamaki et al., 2020). The major carotenoids in WT *E. gracilis* eyespots, i.e. β-carotene, diadinoxanthin, and diatoxanthin (Heelis et al., 1979), were absent or barely detectable in chloroplast-deficient *E. gracilis* eyespots, while zeaxanthin was abundant (Tamaki et al., 2020), suggesting that β-carotene, diadinoxanthin, and diatoxanthin may not be required for eyespot formation and phototaxis. Indeed, the carotenoid species required for phototaxis in euglenophytes remain elusive.

To investigate the association between carotenoid composition and functional eyespot formation in *E. gracilis*, we knocked out the genes putatively involved in carotenoid biosynthesis using clustered regularly interspaced short palindromic repeats (CRISPR)/CRISPR-associated protein 9 (Cas9)-mediated genome editing (Nomura et al., 2019), and generated mutants with different carotenoid compositions. We established that zeaxanthin is required for stable eyespot formation and accurate phototactic responses in *E. gracilis*, in contrast to the sole requirement of β-carotene for eyespot formation and phototaxis in chlorophytes.

## Results

### Identification of putative carotenoid biosynthetic pathway genes in *E. gracilis*

To identify the carotenoid species involved in eyespot formation and phototaxis in *E. gracilis*, we compiled and searched for carotenoid biosynthetic genes. The genes encoding carotenoid biosynthetic enzymes have been characterized, and the carotenoid biosynthetic pathway in *E. gracilis* is diagramed in Supplemental Figure 1 (reviewed in Tamaki et al., 2021a). The genes encoding geranylgeranyl pyrophosphate synthase (*EgcrtE*; Kato et al., 2016), *EgcrtB* (Kato et al., 2016), phytoene desaturase (*EgcrtP1* and *EgcrtP2*; Kato et al., 2019), ζ-carotene isomerase (*EgZ-ISO*; Sugiyama et al., 2020), ζ-carotene desaturase (*EgcrtQ*; Kato et al., 2019), lycopene cyclase (*EgLCY*; Tamaki et al., 2021b), and carotene hydroxylase (*EgCYP97H1*; Tamaki et al., 2019) were genetically and biochemically identified in previous studies (Supplemental Table 1). To obtain the nucleotide sequences of the putative genes encoding prolycopene isomerase (*crtISO*), carotene hydroxylase (*CYP97*), zeaxanthin epoxidase (*ZEP*), and violaxanthin de-epoxidase (*VDE*), we explored a de novo assembly data set of the *E. gracilis* transcriptome (GenBank accession number: GDJR00000000.1) and identified the following putative homologs: *EgcrtISO1*, *EgcrtISO2*, *EgCYP97F2*, *EgZEP1*, *EgZEP2*, *EgZEP3*, *EgVDE1*, and *EgVDE2* (Supplemental Table 1). Although biochemical evidence is not yet available for each of the reactions of EgZEPs and EgVDEs, it should be noted that the proteins encoded by the three *ZEP* and two *VDE* putative homologs may catalyze the epoxidation of diatoxanthin and the de-epoxidation of diadinoxanthin, respectively (Goss and Jakob, 2010; Lavaud et al., 2012; Supplemental Figure 1). Transcripts of the β-carotene ketolase homolog (*crtW* and *crtO*), which are required for echinenone and canthaxanthin biosynthesis (Supplemental Figure 1), and another subclass of carotene hydroxylase (*crtR*, *crtZ*, and *BCH*; Tamaki et al., 2019) were not identified in the *E. gracilis* transcriptome data set. We thus identified 16 genes likely involved in carotenoid biosynthesis in *E. gracilis* as the targets of our genome editing–based mutant analysis.

### Generation of 16 knockout mutants of putative carotenoid biosynthetic genes in *E. gracilis*

**Figure 1:**
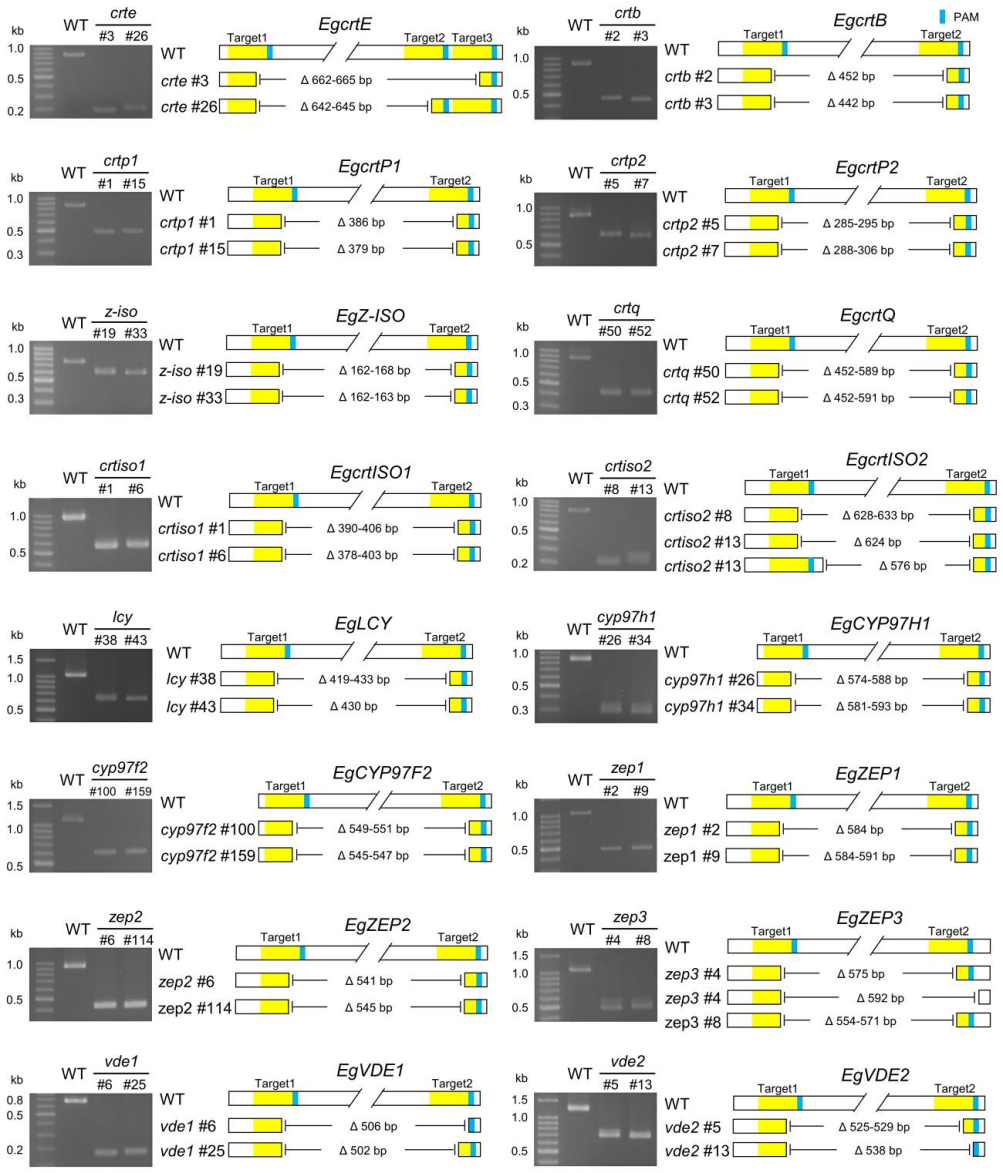
Confirmation of targeted deletions in carotenoid biosynthetic gene knockout mutants. Electrophoresis image (left) and schematic diagram of deletion site (right) of PCR fragments amplified from the WT and each knockout mutant. The gRNA target and PAM sequences are highlighted in yellow and light blue, respectively. The two distinct gRNAs (Targets 1 and 2) were introduced into *E. gracilis* cells. The *crte*#3 and *crte*#26 mutants were generated by introducing different combinations of gRNAs, i.e. target 1/3 and target 1/2, respectively. The nucleotide sequence alignment of PCR fragments is shown in Supplemental Figure 2.

We used our CRISPR/Cas9-mediated genome editing method (Nomura et al. 2019, 2020) to generate knockout mutant lines for each of the 16 putative carotenoid biosynthetic genes listed in Supplemental Table 1. We designed two distinct guide RNA (gRNA) pairs for each target gene to delete approximately 150–600 bp of their genomic regions. Through PCR and amplicon sequencing–based genotyping, we identified knockout mutant lines of the 16 genes (Figure 1 and Supplemental Figure 2). The *EgcrtQ* PCR fragments formed two distinct bands in the WT due to different intron sequences, whereas this region was deleted in the knockout mutants, resulting in a single band. The *EgVDE2* generated two PCR fragments in the mutants, differing by 13 bp of intron sequences outside the genome-edited sites. Some mutants also contained insertions, ranging from 1 to 24 bp (Supplemental Figure 2). We thus established a stable knockout mutant series of carotenoid biosynthetic genes in *E. gracilis*.

### The *crte*, *crtb*, *crtp2*, *crtq*, and *lcy* knockout mutants are deficient in red eyespot formation

**Figure 2:**
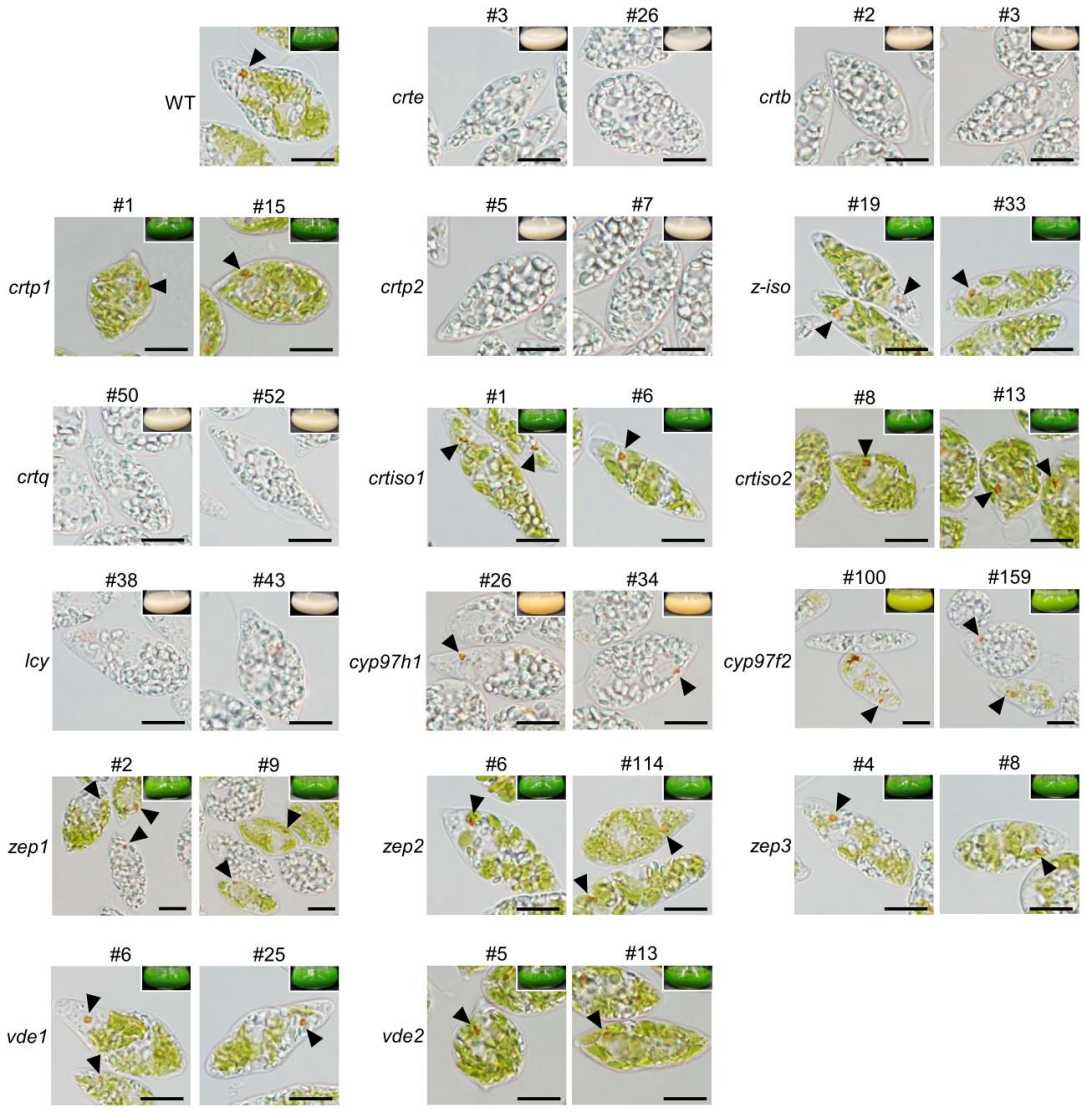
Cell appearances of carotenoid biosynthetic gene knockout mutants. WT and mutant cells were grown in KH medium under continuous light (40 μmol photons m^−2^ s^−1^) at 26°C for 10 days. Arrowheads indicate the eyespot apparatus. Insets are photographic images of culture flasks of each mutant. Scale bars, 10 µm. Magnified images of eyespot-containing areas are shown in Supplemental Figure 3.

We assessed the phenotypes of knockout mutants of the 16 target genes. The *crtp1*, *z-iso*, *crtiso1*, *crtiso2*, *zep2*, *zep3*, *vde1*, and *vde2* mutants were similar in appearance to the WT, with green, well-developed chloroplasts and red eyespots (Figure 2 and Supplemental Figure 3). The *cyp97f2* and *zep1* mutants had intermediate phenotypes, with a mixture of green and colorless cells, despite having homogeneous genotypes, and their cultures ranged from pale to darker green in color. In contrast, the *crte*, *crtb*, *crtp2*, *crtq*, *lcy*, and *cyp97h1* mutants lacked well-developed chloroplasts. Among these mutants, *crte*, *crtb*, *crtp2*, and *crtq* were completely colorless and lacked red eyespots. The *lcy* mutant cultures were pale pink and some *lcy* cells had faint red pigmentation that clearly differed from that of WT eyespots (Figure 2 and Supplemental Figure 3). The *cyp97h1* mutant cultures were orange and the *cyp97h1* mutants formed red eyespots. Taken together, these findings indicate that the β-carotene biosynthetic genes *EgcrtE*, *EgcrtB*, *EgcrtP2*, *EgcrtQ*, and *EgLCY* are required for red eyespot formation in *E. gracilis*.

### Carotenoid composition of the *lcy* and *cyp97* knockout mutants

**Figure 3:**
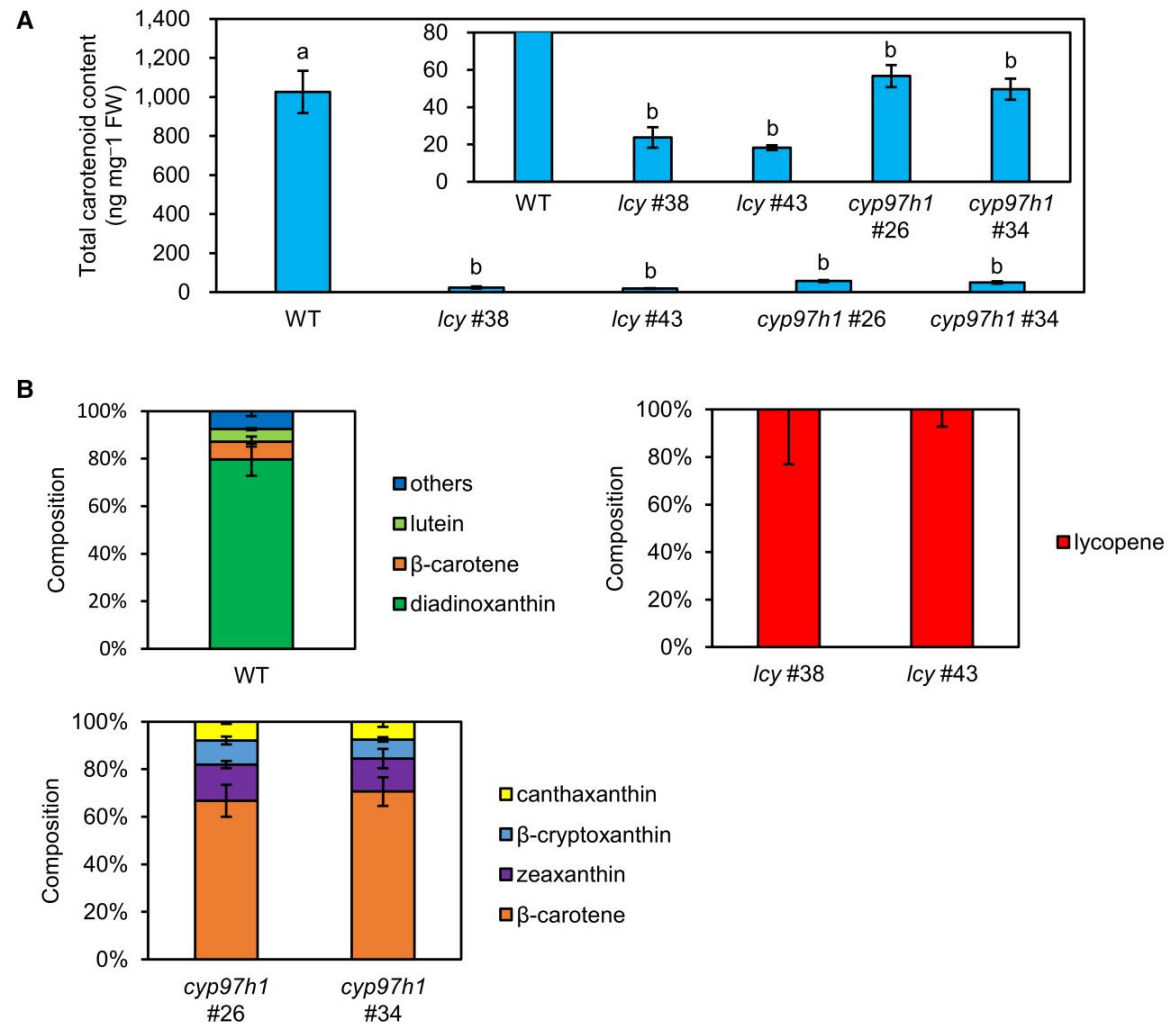
Carotenoid analysis of carotenoid biosynthesis gene knockout mutants. Total carotenoid content (A) and composition (B) of each mutant. Cells were grown in KH medium under continuous light (40 μmol photons m^−2^ s^−1^) at 26°C for 10 days. (A) Inset shows the same graph with a smaller *y*-axis. (B) The composition of other carotenoids in the WT includes neoxanthin (2.4%), violaxanthin (2.2%), antheraxanthin (1.1%), zeaxanthin (1.0%), and diatoxanthin (0.9%). Values are presented as the mean ± SD (*n* = 3). Values with different letters are significantly different from other strains according to the Tukey–Kramer multiple comparison test (*P* < 0.05).

Because the *lcy* and *cyp97h1* mutants both lacked fully developed chloroplasts but differed in eyespot formation (Figure 2 and Supplemental Figure 3), we analyzed their carotenoid composition using liquid chromatography/mass spectrometry (LC/MS). We excluded the *cyp97f2* mutants from further analysis because it was difficult to separately analyze their green and colorless cells, each of which presumably contain markedly different profiles of carotenoids and even other metabolites (Figure 2). The total carotenoid contents of the *lcy* and *cyp97h1* mutants were 1.8%–5.5% that of the WT, which is consistent with the previous observation in the chloroplast-deficient strains (Figure 3A and Tamaki et al., 2020). In the WT cells, we detected neoxanthin, violaxanthin, diadinoxanthin, antheraxanthin, diatoxanthin, zeaxanthin, lutein, and β-carotene, and diadinoxanthin accounted for 79.6% of the total carotenoids (Supplemental Figure 4 and Figure 3B). However, the *lcy* mutants solely accumulated lycopene, suggesting that the lycopene cyclization reaction catalyzed by EgLCY was completely blocked in these mutants. Furthermore, the *cyp97h1* mutants primarily accumulated β-carotene (66.7%–70.6%) and low proportions of zeaxanthin, canthaxanthin, and β-cryptoxanthin. β-Cryptoxanthin and zeaxanthin are downstream hydroxylated compounds of β-carotene produced by carotene hydroxylase (EgCYP97). The presence of these compounds in the *cyp97h1* mutants suggests that EgCYP97H1 and EgCYP97F2 are functionally redundant, and, therefore, that β-carotene hydroxylation is not completely deficient in the *cyp97h1* mutants.

### The *cyp97h1 cyp97f2* double mutants had unstable red eyespot formation

**Figure 4:**
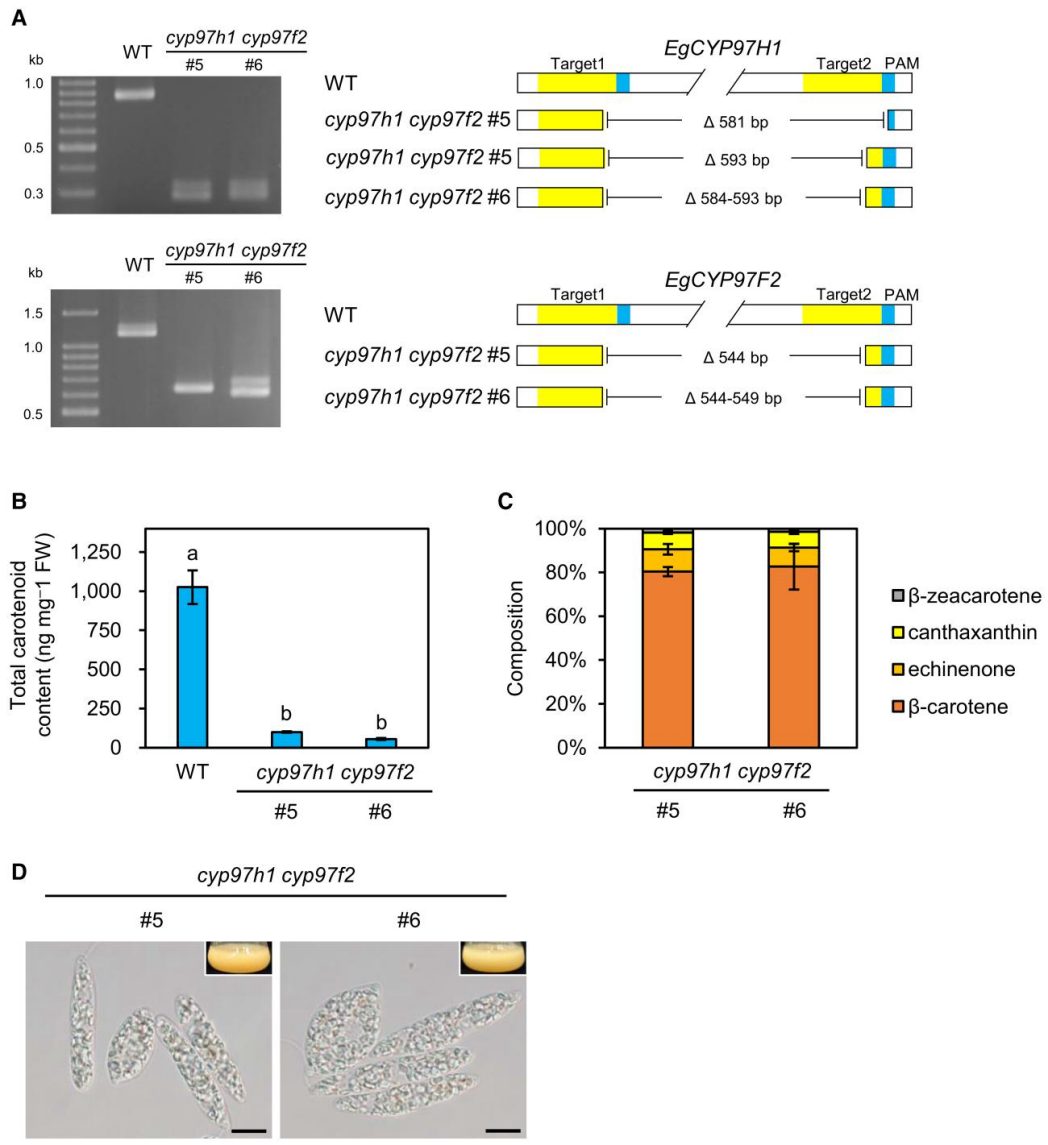
Characterization of *cyp97h1 cyp97f2* double mutants. (A) Electrophoresis image (left) and schematic diagram of deletion site (right) of PCR fragments amplified from the WT and *cyp97h1 cyp97f2* double mutants. The gRNA target and PAM sequences are highlighted. The nucleotide sequence alignment of PCR fragments is shown in Supplemental Figure 5. (B, C) Total carotenoid content (B) and composition (C) of *cyp97h1 cyp97f2* double mutants. Values are presented as the mean ± SD (*n* = 3). Values with different letters are significantly different from other strains according to the Tukey–Kramer multiple comparison test (*P* < 0.05). (D) Cell appearances of *cyp97h1 cyp97f2* double mutants grown in KH medium under continuous light (40 μmol photons m^−2^ s^−1^) at 26°C for 10 days. Arrowheads indicate the eyespot apparatus. Insets are photographic images of culture flasks of each mutant. Scale bars, 10 µm.

To completely knockout β-carotene hydroxylation in *E. gracilis*, we generated *cyp97h1 cyp97f2* double mutants and assessed their phenotype. We induced a deletion in *EgCYP97F2* in the *cyp97h1* cells using *EgCYP97F2* gRNA, and confirmed the edits via genotyping (Figure 4A and Supplemental Figure 5). We examined the carotenoid contents of the *cyp97h1 cyp97f2* double mutants and WT through LC/MS and established that the total carotenoid contents of the *cyp97h1 cyp97f2* double mutants were markedly decreased (Figure 4B). In the *cyp97h1 cyp97f2* double mutants, β-carotene (80.4%–82.6%) was the predominant carotenoid, and echinenone (8.7%–10.2%), canthaxanthin (7.3%–7.5%), and β-zeacarotene (1.4%–1.9%), an intermediate of β-carotene biosynthesis, accounted for small proportions of the total carotenoid contents (Figure 4C and Supplemental Figure 4), indicating that β-carotene hydroxylation was completely blocked in the *cyp97h1 cyp97f2* double mutants. Further, the *cyp97h1 cyp97f2* double mutant cultures were orange, and the cells mostly lacked red eyespots (Figure 4D). However, a faint eyespot-like structure was observed in some cells (Supplemental Figure 6). These results demonstrate that zeaxanthin is critical for stable eyespot formation in *E. gracilis*.

### Phototactic movement of the *lcy* and *cyp97* knockout mutants

**Figure 5:**
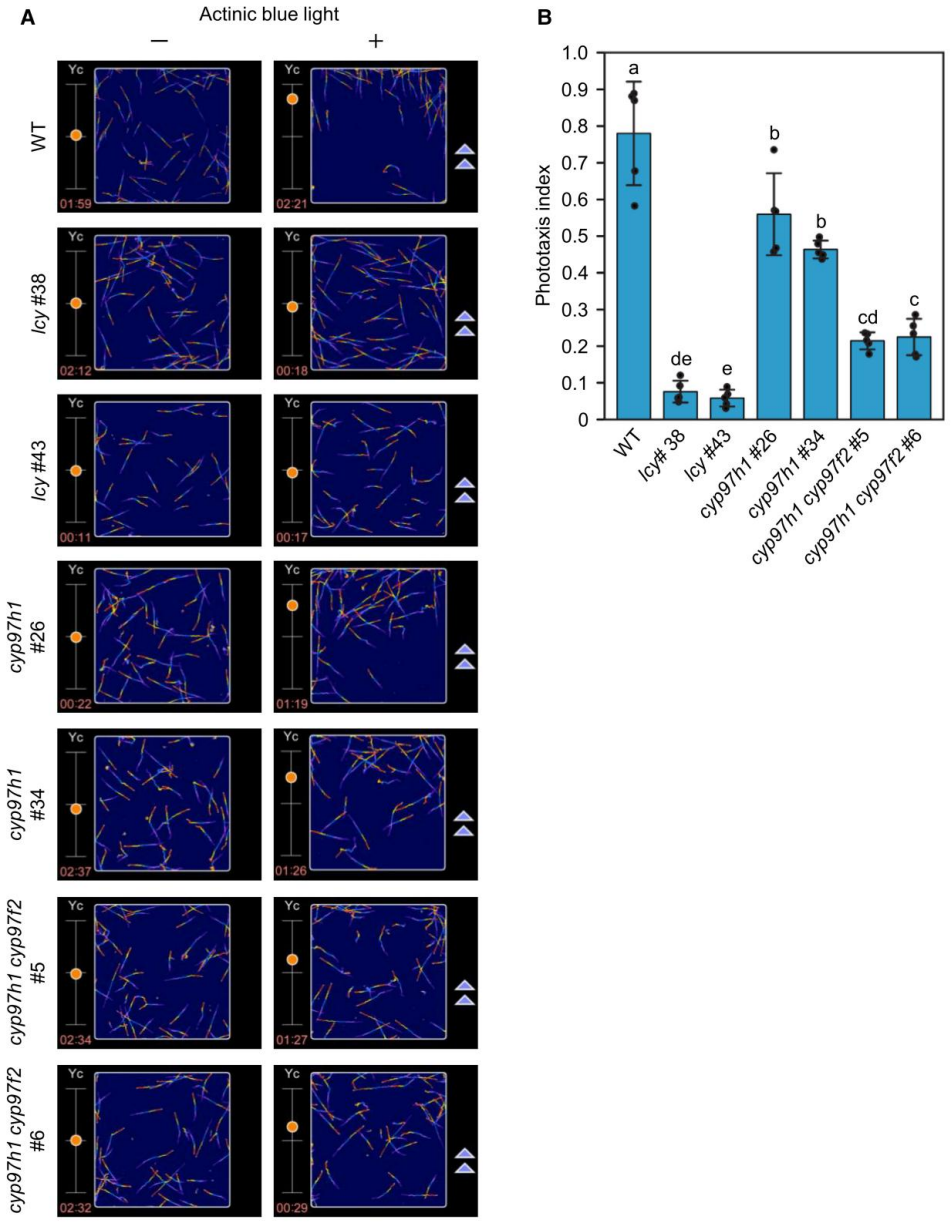
Phototactic analysis of carotenoid biosynthesis gene knockout mutants. Trace images (A) and quantification (B) of the WT and each mutant. Cells in the microchamber were illuminated with actinic blue light at 0 (left column) and 360 (right column) µmol photons m^−2^ s^−1^. (A) Lines indicate subsequent cell traces superimposed for 6.4 s each. Dots (left) and triangles (right) indicate the centroid position of cell traces and the direction of actinic blue light, respectively. (B) The phototaxis index was defined as the light direction amplitude of the centroid position of the cells to the swimming velocity during illumination with actinic blue light. The centroid position of the cells corresponds to the dot in (A). Values are presented as the mean ± SD (*n* = 5). Values with different letters are significantly different from other strains according to the Tukey–Kramer multiple comparison test (*P* < 0.05).

To investigate whether the eyespots observed in the *lcy* and *cyp97* mutants are functional, we assessed their phototactic responses. We previously demonstrated that phototaxis and red eyespot formation are positively associated, regardless of whether chloroplasts develop, in *E. gracilis* (Tamaki et al., 2020). Here, we assessed the phototaxis of the *lcy* and *cyp97* mutants compared with that of the WT using our microchamber device-based motion analysis (Ozasa et al., 2017; Kato et al., 2020). To examine a small number of cells, we used a sodium citrate-free Cramer–Myers (modified CM) medium (Cramer and Myers, 1952) containing ethanol as a carbon source, and confirmed that this culture medium did not affect cell phenotypes (Supplemental Figure 7). The WT and *cyp97h1* mutants, the latter of which have well-formed eyespots but lack fully developed chloroplasts, exhibited phototaxis away from blue light illumination (as shown in Supplemental Movies 1 and 2 and Figure 5A), suggesting that the phototactic response is independent of chloroplast development in *E. gracilis*. In addition, the *lcy* mutants, which formed colorless eyespots, did not exhibit phototaxis (Supplemental Movie 3 and Figure 5A). Moreover, the *cyp97h1 cyp97f2* double mutants, which also had aberrant eyespot formation, had a markedly reduced phototactic response (Supplemental Movie 4 and Figure 5A). Most *cyp97h1 cyp97f2* double mutant cells moved laterally rather than horizontally in the plane of the blue light. To quantify their phototactic response, we traced the centroid position of the cells to measure the amplitude and velocity in their movement away from the light source and computed the phototaxis index. Although the swimming velocities of all mutants were similar to that of the WT (Supplemental Figure 8), the *lcy* mutants and *cyp97h1 cyp97f2* double mutants had a significantly lower phototaxis index than the WT and *cyp97h1* single mutants (Figure 5B). Notably, all mutants exhibited photoshock responses against extremely strong blue light illumination (Supplemental Figure 9), indicating that the photoreception of these strains was functional regardless of their phototactic abilities. These results demonstrate that the β-carotene biosynthetic pathway and zeaxanthin are required for accurate phototaxis in *E. gracilis*.

## Discussion

### Pathway-scale gene knockout provided a detailed understanding of carotenoid biosynthesis in *E. gracilis*

Here, we aimed to investigate the relationship between carotenoid composition and functional eyespot formation in *E. gracilis*. Reverse genetics studies in *E. gracilis* have mainly relied on RNAi-based methods with electroporation of double-stranded RNAs (e.g. Iseki et al., 2002, Tamaki et al., 2019). However, RNAi-based gene knockdown effects are transient and often result in a mixture of knockdown and WT cells. To overcome these undesired effects, we used a CRISPR/Cas9-mediated genome editing method (Nomura et al., 2019) to generate stable knockout lines of each of the 16 putative carotenoid biosynthetic genes and validated their role in eyespot formation in *E. gracilis*.

Among the knockout lines, the *crte*, *crtb*, *crtp2*, and *crtq* mutants completely lacked fully developed chloroplasts and were colorless (Figure 2). This phenotype was also observed in *EgcrtB* and *EgLCY* RNAi lines, which had a significant reduction of carotenoid and chlorophyll contents (Kato et al., 2017; Tamaki, et al. 2021b). *EgLCY* knockdown perturbed the antioxidant system (i.e. the ascorbate–glutathione cycle), leading to reactive oxygen species (ROS) accumulation (Tamaki, et al., 2021b). Therefore, the aberrant chloroplast development in these lines may be caused by excessive ROS accumulation due to the carotenoid deficiency, as was also observed in other photosynthetic organisms such as Arabidopsis (*Arabidopsis thaliana*; Kim and Apel, 2013; Zhao et al., 2018) and *C. reinhardtii* (McCarthy et al., 2004). A complementation experiment in *Escherichia coli* demonstrated that EgCrtP1 and EgCrtP2 had phytoene desaturase activity (Kato et al., 2019), but in our study, *EgcrtP1* knockout caused no visible phenotypic change, while *EgCrtP2* knockout caused the colorless phenotype (Figure 2). In *E. gracilis* transcriptome data, *EgcrtP1* expression was 10-fold lower than that of *EgcrtP2* (Kato et al., 2019). These findings suggest that EgCrtP2 may be the dominant phytoene desaturase in *E. gracilis*. The *z-iso*, *crtiso1*, and *crtiso2* mutant cells were green, like the WT (Figure 2). These results may be explained by the fact that the *cis* form of carotenes is isomerized by Z-ISO and CrtISO in the dark, whereas they are non-enzymatically photoisomerized in the light (Breitenbach and Sandmann, 2005; Sugiyama et al., 2020).

The *lcy* mutant, which is deficient in the conversion of lycopene to β-carotene or α-carotene, was colorless, had a marked reduction in total carotenoid content, and only accumulated lycopene (Figures 2 and 3). The colorless phenotype of this mutant was consistent with the results of a previous study of RNAi-based *EgLCY* knockdown mutants (Tamaki et al., 2021b). *EgLCY* encodes a lycopene β-cyclase, which forms β-rings at both ends of lycopene to produce β-carotene (Tamaki, et al., 2021b). The specific accumulation of lycopene in the *lcy* mutants suggests that EgLCY solely functions as a lycopene cyclase in *E. gracilis*. However, lutein, a hydroxylated carotenoid of α-carotene produced by β- and ε-cyclization of lycopene (Supplemental Figure 1; Tamaki et al., 2021a), was detected in the WT (Figure 3 and Supplemental Figure 4). These observations raise the question of how *E. gracilis* biosynthesizes α-carotene and subsequently hydroxylates it to lutein. One possibility is that EgLCY catalyzes both β- and ε-cyclization in *E. gracilis*, in the same manner as the LCYs in *Dunaliella bardawil* (Liang et al., 2019). However, because α-carotene and lutein were undetectable even in the *cyp97h1* single mutants and *cyp97h1 cyp97f2* double mutants (Supplemental Figure 4) as well as in the WT grown under different culture conditions (Tamaki et al., 2020, 2021b), its lycopene ε-cyclization activity may be much lower than its β-cyclization activity in *E. gracilis*.

The cellular, metabolic, and phototactic phenotypes of the *cyp97h1* single mutants and *cyp97h1 cyp97f2* double mutants suggest that CYP97H1 and CYP97F2 are functionally redundant in β-carotene hydroxylation, which is critical for phototaxis in *E. gracilis*. Contrary to the *cyp97h1* single mutant phenotype, which accumulated zeaxanthin (Figure 3B) and had a less decreased phototaxis index (Figure 5B), the *cyp97h1 cyp97f2* double mutants were deficient in zeaxanthin and had a significantly reduced phototaxis index (Figure 5B), clearly indicating the functional redundancy of EgCYP97H1 and EgCYP97F2. However, the *cyp97h1 cyp97f2* double mutants and *cyp97h1* single mutants were colorless (Figures 2 and 4D), suggesting that chloroplast development in *E. gracilis* may depend on a threshold level of accumulated xanthophylls such as zeaxanthin. Thus, the *cyp97f2* mutants may be able to exceed such a threshold, but the *cyp97h1* single mutants and *cyp97h1 cyp97f2* double mutants may not. Furthermore, the unstable chloroplast development observed in *cyp97f2* cells implies that EgCYP97H1 may be the dominant β-carotene hydroxylation enzyme in *E. gracilis* under our experimental conditions. Analysis of the *cyp97h1 cyp97f2* double mutants revealed that β-carotene hydroxylation has a critical role in functional eyespot formation and accurate phototaxis in *E. gracilis*, contrary to the finding that β-carotene is primarily responsible for these functions in chlorophyte species such as *C. reinhardtii* (Kreimer, 2009; Pick et al., 2019).

### The divergent regulation of functional eyespot formation between euglenophytes and chlorophytes

Our carotenoid biosynthesis pathway-scale gene knockout analysis in *E. gracilis* revealed the hierarchal relationship between red eyespot formation and the function of the eyespot in phototaxis. We can summarize our findings as follows: (1) the *lcy* mutants lacked the red eyespot apparatus (Figure 2 and Supplemental Figure 3), did not accumulate β-carotene and its derivatives (Figure 3), and did not exhibit phototaxis (Figure 5 and Supplemental Movie 3); (2) the *cyp97h1 cyp97f2* double mutants rarely formed red eyespots (Supplemental Figure 6), did not accumulate hydroxylated carotenoids (Figure 4C), and had a significantly lower phototaxis index than the WT, but a higher phototaxis index than the *lcy* mutants (Figure 5); and (3) the *cyp97h1* mutants formed red eyespots (Figure 2 and Supplemental Figure 3), accumulated β-carotene and its downstream hydroxylated compounds such as β-cryptoxanthin and zeaxanthin (Figure 3), yet exhibited a higher phototaxis index than the *cyp97h1 cyp97f2* double mutants (Figure 5 and Supplemental Movie 2). Taken together, our results revealed that zeaxanthin is required for eyespot formation and accurate phototaxis in *E. gracilis*.

**Figure 6:**
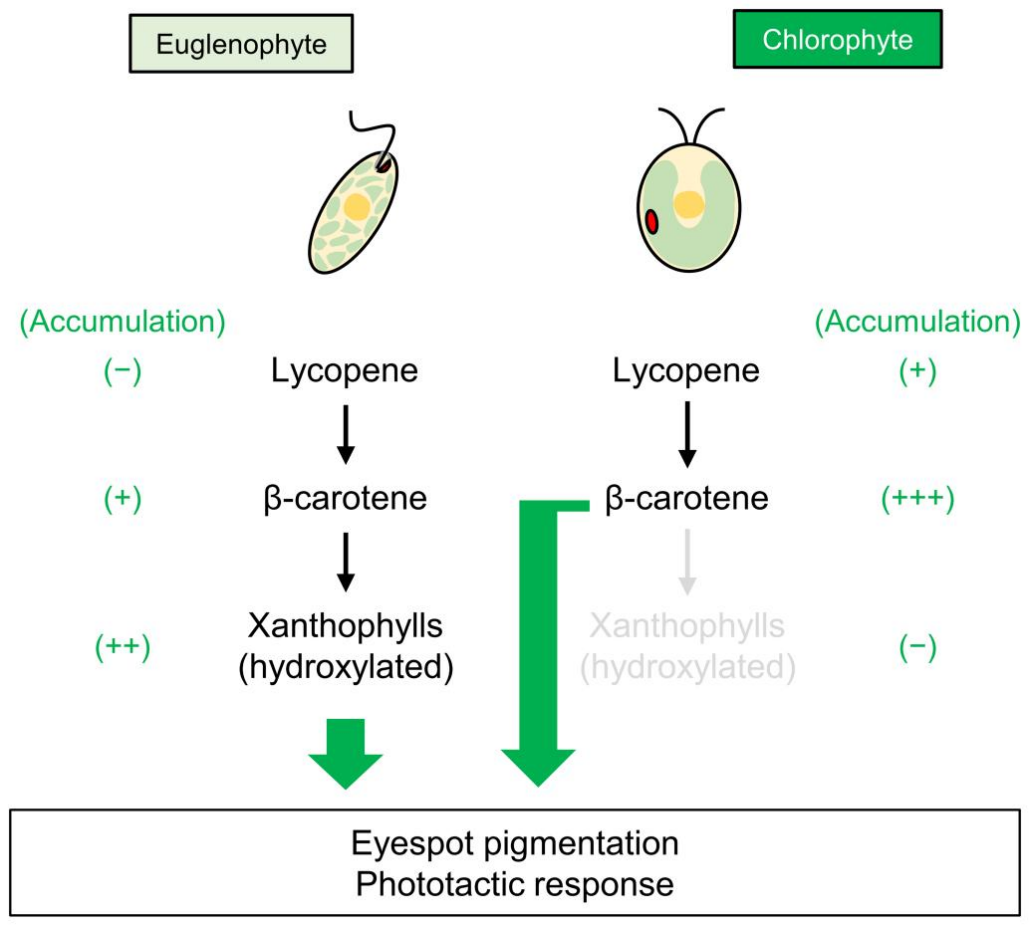
Differences in the eyespot carotenoids of flagellated microalgae. This study demonstrated that xanthophylls dominate, and β-carotene partially contributes to functional eyespot formation and phototaxis in *E. gracilis*, whereas lycopene has no contribution to eyespot formation. In contrast to euglenophytes, chlorophytes form β-carotene-rich eyespots. Some chlorophytes with a less-pigmented chloroplast accumulate lycopene in the eyespot apparatus (Kreimer, 2009). The existence of xanthophylls in the chlorophyte eyespot has not been documented.

The difference in the carotenoid species required for eyespot formation between *E. gracilis* and chlorophytes such as*C. reinhardtii* indicates that eyespot development in members of the microalgal phylum underwent distinct evolutionary paths (Figure 6). In chlorophytes, eyespot apparatuses are derived from β-carotene-rich plastoglobules formed inside the chloroplast (Schmidt et al., 2006; Pick et al., 2019). *C. reinhardtii* and *Pyramimonas parkae* eyespots are mainly composed of β-carotene and a small amount of α- and γ-carotene. In the *C. reinhardtii* eyespot, the cis-β-carotene and trans-β-carotene ratio is skewed to the cis form, which structurally prevents β-carotene hydroxylation to zeaxanthin (Kreimer, 2009). Moreover, β-carotene was depleted in the eyespot-lacking *C. reinhardtii* mutant *lts1-211* (Ueki et al., 2016). These findings suggest that β-carotene alone is critical for functional eyespot formation in chlorophytes. However, β-carotene, diadinoxanthin, and diatoxanthin (which account for more than 60% of the total carotenoids), and some unknown xanthophylls were observed in the eyespot fraction isolated from light-grown *E. gracilis* (Heelis et al., 1979). A recent study with chloroplast-deficient *E. gracilis* strains (SM-ZK, *cl1*, and *cl3*) that form functional eyespots revealed that they accumulate zeaxanthin as the major carotenoid, but do not accumulate diadinoxanthin and diatoxanthin (Tamaki et al., 2020). These findings and our genetics-based findings indicate that *E. gracilis* eyespots are mainly composed of xanthophylls such as zeaxanthin and diadinoxanthin.

What is the difference between β-carotene and xanthophylls? Xanthophylls are components of the xanthophyll cycle, which is the photoprotective mechanism that operates through non-photochemical quenching (NPQ) in chloroplasts. In chlorophytes, violaxanthin is de-epoxidized to zeaxanthin under high-light conditions, and the reverse reaction occurs under low-light or dark conditions (the violaxanthin cycle) to optimize the light-harvesting efficiency of photosystem II (Goss and Jakob, 2010). In diatoms, haptophytes, and dinoflagellates, the interconversion of diadinoxanthin and diatoxanthin (the diadinoxanthin cycle) modulates NPQ (Goss and Jakob, 2010). Although it was assumed that diadinoxanthin was mainly involved in the xanthophyll cycle in *E. gracilis* cells because of the large pool of diadinoxanthin present (Figure 3B; Kato et al., 2017; Doege et al., 2000), the observation that the de-epoxidation of diadinoxanthin to diatoxanthin could not be promoted by light illuminations (20 min, 50, 300, or 1500 μmol m^−2^ s^−1^) that induced time-dependent fluorescence quenching suggested that NPQ may not depend on the xanthophyll cycle in *E. gracilis* (Doege et al. 2000).

Given the importance of zeaxanthin in functional eyespot formation in *E. gracilis*, our findings led us to hypothesize that zeaxanthin may function in the photoprotective machinery of the eyespot. To rapidly respond to fluctuating light environments, photoreceptors would be equipped with some sort of photoprotective machinery that optimizes the light environment and avoids damage by excess light (Allorent and Petroutsos, 2017). *Euglena gracilis* regulates phototaxis through photoactivated adenylyl cyclase, a flavin chromophore-containing photoreceptor located in the paraflagellar body near the base of the major flagellum, close to the eyespot apparatus in the cytosol (Häder and Iseki, 2017; Hammond et al., 2021). Flavin chromophore photoreceptors generate ROS in a light-dependent manner (Consentino et al., 2015; Arthaut et al., 2017). These ROS may damage the eyespot apparatus in *E. gracilis*. Therefore, carotenoids localized to the eyespot could directly scavenge the ROS, or in the case of zeaxanthin, the xanthophyll cycle may provide rapid photoprotection to avoid light-induced damage to the eyespot apparatus. In contrast to the situation in euglenophytes, the eyespot apparatus is localized to the chloroplast in chlorophytes (Kreimer, 2009; Pick et al., 2019). The eyespot in chlorophytes may thus outsource its photoprotective machinery to the chloroplast.

Although our study demonstrates that zeaxanthin is required for the stable formation of functional eyespots in *E. gracilis*, the molecular function of zeaxanthin in *E. gracilis* remains elusive. In the context of the xanthophyll cycle, the enzymatic functions of VDE and ZEP homologs need to be clarified in *E. gracilis*. Recently, Kato et al. (2017) examined the effects of prolonged continuous light intensities (7 days, 27, 55, 240, 460, or 920 μmol m^−2^ s^−1^) on carotenoid content in *E. gracilis*, and demonstrated that illumination at 920 μmol m^−2^ s^−1^ significantly increased the cellular content of β-carotene, diadinoxanthin, and diatoxanthin, as well as the diatoxanthin/diadinoxanthin ratio (Tukey's multiple range test, *P* < 0.05), suggesting that prolonged continuous light stress may induce de-epoxidation by enzymes involved in the xanthophyll cycle such as VDE in *E. gracilis* (Supplemental Figure 1). However, in a recent subcellular-level proteome analysis, VDE enzyme was not detected in *E. gracilis* plastids (Novák Vanclová et al., 2020), implying that enzymes involved in the xanthophyll cycle might function in pigment-enriched cellular compartments such as the eyespot apparatus in *E. gracilis*.

## Materials and methods

### Strain and culture conditions

*Euglena gracilis* strain Z (NIES-48) was grown in Koren–Hutner (KH) medium (pH 3.5; Koren and Hutner, 1967) under continuous light (40 μmol photons m^−2^ s^−1^) in a NC-411PFD-H growth chamber (Nippon Medical & Chemical Instruments, Osaka, Japan) at 26°C with a Shake-LR2 rotary shaker (120 rpm; Taitec, Aichi, Japan). The light intensity was measured using an HD2302.01 portable luxmeter and LP471PAR probe (400–700 nm; Delta Ohm, Veneto, Italy).

### CRISPR/Cas9-mediated genome editing

To knockout the 16 carotenoid biosynthetic genes present in *E. gracilis* (Supplemental Table 1), template DNA for gRNA preparation was amplified using primer a (including the target sequence), primer b, and primer c, and purified. All primer sequences used in this study and gRNA target sequences are listed in Supplemental Tables 2 and 3, respectively. Each gRNA was synthesized from a PCR fragment using a CUGA7 gRNA Synthesis Kit (Nippon Gene, Tokyo, Japan), following the manufacturer's instructions. Each 4 μg of gRNA solution was mixed with 50 pmol of Alt-R S.p. Cas9 Nuclease V3 (Integrated DNA Technologies, IA, United States of America) and incubated at room temperature for 15 min. The ribonucleoprotein (RNP) complex was electroporatically introduced into *E. gracilis* cells following the method described in our previous study (Nomura et al., 2020), and the cells were then grown in KH medium (pH 5.5) for 7 days. Two types of RNP complexes targeting different sequences were used to generate deletions in the genomic sequence in each gene.

### Isolation of knockout cell lines

Individual genome-edited cells were sorted into a 96-well plate, with each well containing 200 μL of modified CM:KH (4:1 v/v) medium (pH 3.5), using a MoFlo XDP fluorescence-activated cell sorter (Beckman Coulter, CA, United States of America), following the method described in our previous study (Yamada et al., 2016). After culture for 12 days, genomic DNA was extracted from the cloned cell lines using a Kaneka Easy DNA Extraction Kit v.2 (Kaneka, Osaka, Japan). To confirm the deletion of targeted genomic sequences, DNA fragments containing the genome-edited sites were amplified using gene-specific primers (Supplemental Table 2) and analyzed by gel electrophoresis on a 2% (w/v) agarose gel. The amplified DNA fragments were cloned into the pJET1.2/blunt vector (Thermo Fisher Scientific, MA, United States of America) and Sanger sequenced.

### Microscopy analysis

Microscopy images were obtained using an Olympus BX51 upright microscope with a 100× objective lens (Olympus, Tokyo, Japan).

### Carotenoid determination

Carotenoid extraction and LC/MS analysis were performed following the method described by Tamaki et al. (2020).

### Photomovement analysis

Confinement of *E. gracilis* cells grown in modified CM medium containing 0.1% (v/v) ethanol in a square microchamber (2.5 mm each side; 100 μm depth) and photomovement analysis were performed following the method described by Tamaki et al. (2020).

### Statistical analysis

All data are presented as the mean ± SD. Differences between WT and mutant lines were assessed using the Tukey–Kramer multiple comparison test and considered to be statistically significant at *P* < 0.05.

### Accession numbers

Sequence data from this article can be found in the GenBank/EMBL data libraries under accession numbers described in Supplemental Table 1.

## Supplemental data

The following materials are available in the online version of this article.

**Supplemental Figure S1.** The carotenoid biosynthetic pathway in *E. gracilis*.

**Supplemental Figure S2.** The nucleotide sequence alignment of PCR fragments amplified from the wild-type (WT) and each knockout mutant.

**Supplemental Figure S3.** Images of the eyespots of each knockout mutant.

**Supplemental Figure S4.** LC/MS analysis of carotenoids extracted from the wild-type (WT) and each mutant.

**Supplemental Figure S5.** The nucleotide sequence alignment of PCR fragments amplified from the wild-type (WT) and *cyp97h1 cyp97f2* double mutants.

**Supplemental Figure S6.** Rarely observed red eyespots of *cyp97h1 cyp97f2* double mutants.

**Supplemental Figure S7.** Cell appearances of carotenoid biosynthetic gene knockout mutants.

**Supplemental Figure S8.** The swimming velocities of carotenoid biosynthetic gene knockout mutants during phototactic analysis.

**Supplemental Figure S9.** Photoshock analysis of carotenoid biosynthesis gene knockout mutants.

**Supplemental Table S1.** List of putative carotenoid biosynthetic genes knocked out in *E. gracilis*.

**Supplemental Table S2.** Primer sequences used in this study.

**Supplemental Table S3.** Target sequences for gRNA synthesis.

**Supplemental Movie S1.** Movement tracks of wild-type (WT) *E. gracilis* illuminated with blue light at 0 or 360 µmol photons m^−2^ s^−1^.

**Supplemental Movie S2.** Movement tracks of *E. gracilis cyp97h1* mutants illuminated with blue light at 0 or 360 µmol photons m^−2^ s^−1^.

**Supplemental Movie S3.** Movement tracks of *E. gracilis lcy* mutants illuminated with blue light at 0 or 360 µmol photons m^−2^ s^−1^.

**Supplemental Movie S4.** Movement tracks of *E. gracilis cyp97h1 cyp97f2* double mutants illuminated with blue light at 0 or 360 µmol photons m^−2^ s^−1^.

## Supplementary Material

kiad001_Supplementary_Data
