# Supplementary material for: Zeaxanthin is required for eyespot formation and phototaxis in Euglena gracilis
Source: Plant Physiol. 2023 Jan 6;191(4):2414–26. doi: 10.1093/plphys/kiad001 (PMC10069888; doi:10.1093/plphys/kiad001)
Supplement: kiad001_Supplementary_Data [file kiad001_supplementary_data.zip › CarGE_SupplementaryTable_6.pdf]

Supplemental Table S1. List of putative carotenoid biosynthetic genes knocked out in *E. gracilis*

Gene	Accession number	Function/Annotation	E-value [Uniprot ID of query]	Reference
EgcrtE	LC700283<br>LC700284<br>LC700285	geranylgeranyl pyrophosphate synthase	—	Kato et al., 2016
EgcrtB	LC700286<br>LC700287	phytoene synthase	—	Kato et al., 2016
EgcrtP1	LC700288	phytoene desaturase	—	Kato et al., 2019
EgcrtP2	LC700289<br>LC700290	phytoene desaturase	—	Kato et al., 2019
EgZ-ISO	LC700291<br>LC700292<br>LC700293	ζ-carotene isomerase	—	Sugiyama et al., 2019
EgcrtQ	LC700294<br>LC700295	ζ-carotene desaturase	—	Kato et al., 2019
EgcrtISO1	LC700296<br>LC700297	prolycopene isomerase	3e-60<br>[CRTSO_SOLLC]	—
EgcrtISO2	LC700298<br>LC700299	prolycopene isomerase	2e-43<br>[CRTSO_SOLLC]	—
EgLCY	LC700300	lycopene cyclase	—	Tamaki et al., 2021b
EgCYP97H1	LC700301<br>LC700302	β-carotene hydroxylase	—	Tamaki et al., 2019
EgCYP97F2	LC700303<br>LC700304	β-carotene hydroxylase	1e-105 [LUT5 _ARATH]	—
EgZEP1	LC700305	zeaxanthin epoxidase	5e-68	—

		(diatoxanthin epoxidase)	[ZEP_ARATH]	
EgZEP2	LC700306	zeaxanthin epoxidase (diatoxanthin epoxidase)	5e-11 [ABA2_PRUAR]	—
EgZEP3	LC700307 LC700308	zeaxanthin epoxidase (diatoxanthin epoxidase)	7e-16 [A0A2V0P950_9CHLO]	—
EgVDE1	LC700309	violaxanthin de-epoxidase (diadinoxanthin de-epoxidase)	1e-47 [VDE_ARATH]	—
EgVDE2	LC700310 LC700311	violaxanthin de-epoxidase (diadinoxanthin de-epoxidase)	2e-84 [I1K4S1_SOYBN]	—

Supplemental Table S2. Primer sequences used in this study.

Primer	Sequence
Primer a	5'-CTAATACGACTCACTATAG-(20 bp target sequence)-GTTTTAGAGCTAGAAATAGCAAGTTAAAATA-3'
Primer b	5'-AAAGCACCGACTCGGTGCCACTTTTTCAAGTTGATAACGGACTAGCCTTATTTTAACTTGCTATTTCTA-3'
Primer c	5'-AAAAGCACCGACTCGGTGCC-3'
EgcrtE-F	5'-AATACCCTTCTGTCTCATCCGAC-3'
EgcrtE-R	5'-CAAGTGCATCTCACAAACACTGAT-3'
EgcrtB-F	5'-AGACGAAGACCTTCAATGCACAG-3'
EgcrtB-R	5'-AAGCAATGCTATGGCTGGATCTT-3'
EgcrtP1-F	5'-AAGCCAGAACAAATAGATCAACCAC-3'
EgcrtP1-R	5'-TACCTGGAAGAGGTTCATCATGTT-3'
EgcrtP2-F	5'-CAATTGCATAATGGAACCTCTTCT-3'
EgcrtP2-R	5'-CCATGGCCAAATCTTCTTGTG-3'
EgZ-ISO-F	5'-GACATCCACCACTCCATATGTTG-3'
EgZ-ISO-R	5'-TTCTATTCGGTGTGCTGCAAACT-3'
EgcrtQ-F	5'-TTCAACATTGGTGTGTTTTGTGC-3'
EgcrtQ-R	5'-GTTCAATCATCCTCACAACAGTGA-3'
EgcrtISO1-F	5'-TGTTATATTTTGGGAGTTGTCTCGC-3'
EgcrtISO1-R	5'-GCAAACATTGGAACACATTTGC-3'
EgcrtISO2-F	5'-AAGAAATTTCCTGCTCGTGATAC-3'
EgcrtISO2-R	5'-CTTCACATCCACTTTCATTCACAC-3'
EgLCY-F	5'-TTTCCATGTTGTATAATCAGCACTT-3'
EgLCY-R	5'-ATCCATAGTCATCCGTAGTTTCAGG-3'
EgCYP97H1-F	5'-TTTATCCAGGTACAGCACAGTCG-3'
EgCYP97H1-R	5'-TTACAGAGAAATTTGGCTTGCTG-3'
EgCYP97F2-F	5'-GTCACATAACACTGCTGCTGTTT-3'
EgCYP97F2-R	5'-ATTGGAGCCAAAACATATGCAC-3'
EgZEP1-F	5'-CTGGGAGTTCAGGTACCGGTAAT-3'
EgZEP1-R	5'-TTATCCCAACTGCAATTTTAGGCA-3'
EgZEP2-F	5'-ACACCAGCACGTTGAACAGAATA-3'
EgZEP2-R	5'-ATTCCTGTGTATCCTGTGAAGTCC-3'
EgZEP3-F	5'-TCGTGTAGAAACTGTCCCAAAATG-3'
EgZEP3-R	5'-AACTATGGCAATGGATCTTTGGTC-3'
EgVDE1-F	5'-CACTCTCACTCAAATACATGTGGC-3'
EgVDE1-R	5'-AAACGTGAGATTCGGGATGTTG-3'

EgVDE2-F	5’-ATGGTGAAGAATAACCAGCTGGAT-3’
EgVDE2-R	5’-CTATGCTTCGAGTGGTGTTCAAAT-3’

Supplemental Table S3. Target sequences for gRNA synthesis.

Gene	Sequence
EgcrtE-1	5'-ACCCCGTGGCATCCGCCTCC-3'
EgcrtE-2	5'-CCTCTCCACAGGAGGCGTTG-3'
EgcrtE-3	5'-ACGCTTGTTGCAAATGAGCC-3'
EgcrtB-1	5'-TAATGAGGTCGAGAAGATTA-3'
EgcrtB-2	5'-CCGTCGCACGGATGAGATTG-3'
EgcrtP1-1	5'-GGATACCGACAACTTCCGCG-3'
EgcrtP1-2	5'-GCATCTCCCCATTGTCCTCG-3'
EgcrtP2-1	5'-CAACCATTTGCATCTCACCC-3'
EgcrtP2-2	5'-GCAGCCAAGTTCTCGTCAGG-3'
EgZ-ISO-1	5'-GCTGCCCATTACCTCTTTAT-3'
EgZ-ISO-2	5'-GCAAGTTCGCCACGGGTCGA-3'
EgZ-ISO-3	5'-GCAAGTTCGCCATGGGTCGA-3'
EgcrtQ-1	5'-GGAAAGCACCTCCGTCGACC-3'
EgcrtQ-2	5'-GCTGGCGGCGGACGGCAAGA-3'
EgcrtISO1-1	5'-ATCATAGACGCGCTGGTCCG-3'
EgcrtISO1-2	5'-GCGACGGTGTGGGACACGTT-3'
EgcrtISO2-1	5'-CGCGGCCTTCAGCGGTGCGC-3'
EgcrtISO2-2	5'-CGTGACAGCACCGTTCGCGG-3'
EgLCY-1	5'-AGGCCACGCTGGGCATCCGC-3'
EgLCY-2	5'-GAGGCGACCCGACTTCCGCG-3'
EgCYP97H1-1	5'-GCGGTGTTCAATTATGAGTT-3'
EgCYP97H1-2	5'-TGTGTACGCTGTGATGCGTG-3'
EgCYP97F2-1	5'-GATCTACGCCGACATGCCGT-3'
EgCYP97F2-2	5'-GTATTTTGAGAACTTCGGCT-3'
EgZEP1-1	5'-AATGATGCCCAACCTCGGCC-3'
EgZEP1-2	5'-GGCTGCTTCAGGAGTACTAC-3'
EgZEP2-1	5'-GGGTATCGTGTGCCGCTTCG-3'
EgZEP2-2	5'-CAGCGGAGATCAACGCGGAC-3'
EgZEP3-1	5'-GGCATGACAATCCTGACCCT-3'
EgZEP3-2	5'-CGCCCACAACGTCCGCATCA-3'
EgVDE1-1	5'-GCCACACGTTTACGGTGGAC-3'
EgVDE1-2	5'-CGTCGACCAGTCGTGGGTCC-3'
EgVDE2-1	5'-GGTGGCCTGTATGCAGTCCC-3'
EgVDE2-2	5'-CATCACCTCGTATGAGAATG-3'

Three types of gRNA for *EgcrtE* were used as described in Figure 3. *EgZ-ISO-2* and *EgZ-ISO-3* gRNAs include a 1-bp substitution, and thus *E. gracilis* was treated with half the amount (2 μg each) of *EgZ-ISO-2* and *EgZ-ISO-3* gRNAs to knock out *EgZ-ISO*.
