# Supplementary material for: Zeaxanthin is required for eyespot formation and phototaxis in Euglena gracilis
Source: Plant Physiol. 2023 Jan 6;191(4):2414–26. doi: 10.1093/plphys/kiad001 (PMC10069888; doi:10.1093/plphys/kiad001)
Supplement: kiad001_Supplementary_Data [file kiad001_supplementary_data.zip › Supplemental Data.pdf]

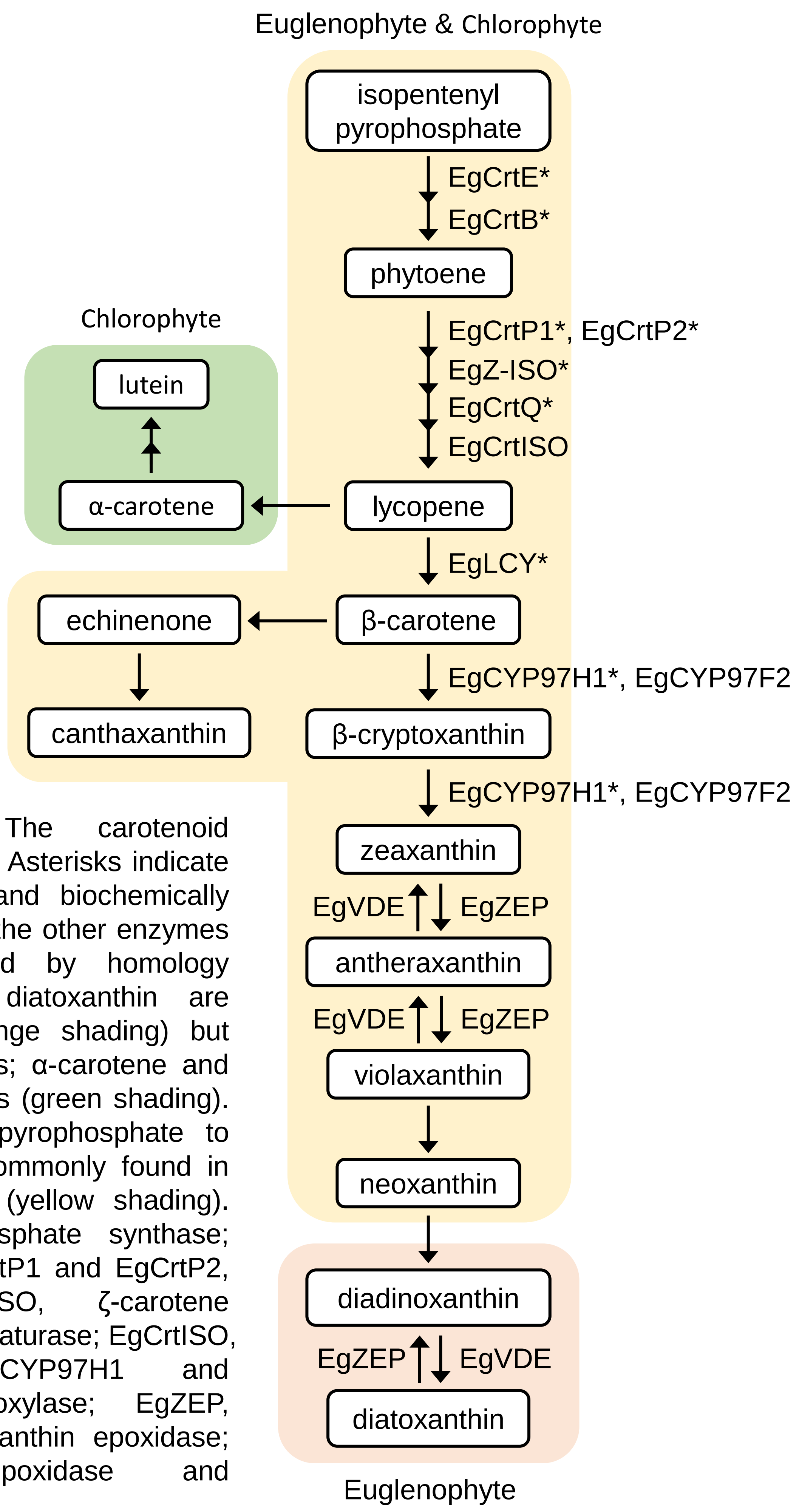

Supplemental Figure S1. The carotenoid biosynthetic pathway in *E. gracilis*. Asterisks indicate enzymes that were genetically and biochemically characterized in previous studies; the other enzymes are putative enzymes identified by homology searches. Diadinoxanthin and diatoxanthin are abundant in euglenophytes (orange shading) but were not detected in chlorophytes; α-carotene and lutein are abundant in chlorophytes (green shading). The pathway from isopentenyl pyrophosphate to neoxanthin or canthaxanthin is commonly found in euglenophytes and chlorophytes (yellow shading). EgCrtE, geranylgeranyl pyrophosphate synthase; EgCrtB, phytoene synthase; EgCrtP1 and EgCrtP2, phytoene desaturase; EgZ-ISO, ζ-carotene isomerase; EgCrtQ, ζ-carotene desaturase; EgCrtISO, prolycopene isomerase; EgCYP97H1 and EgCYP97F2, β-carotene hydroxylase; EgZEP, zeaxanthin epoxidase and diatoxanthin epoxidase; EgVDE, violaxanthin de-epoxidase and diadinoxanthin de-epoxidase.

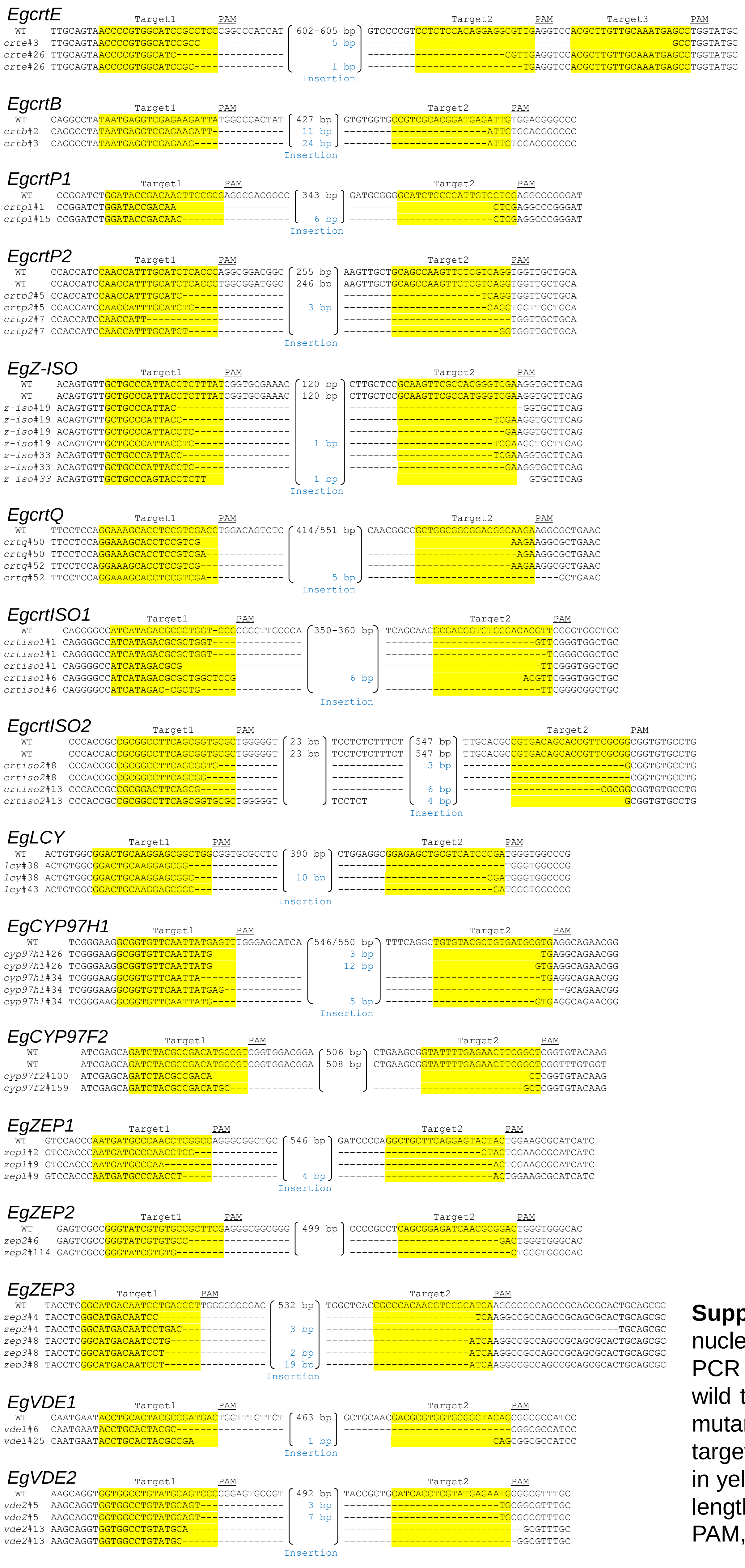

Supplemental Figure S2. The nucleotide sequence alignment of PCR fragments amplified from the wild type (WT) and each knockout mutant. The guide RNA (gRNA) target sequences are highlighted in yellow. Blue letters represent the length of the insertion sequence. PAM, protospacer adjacent motif.

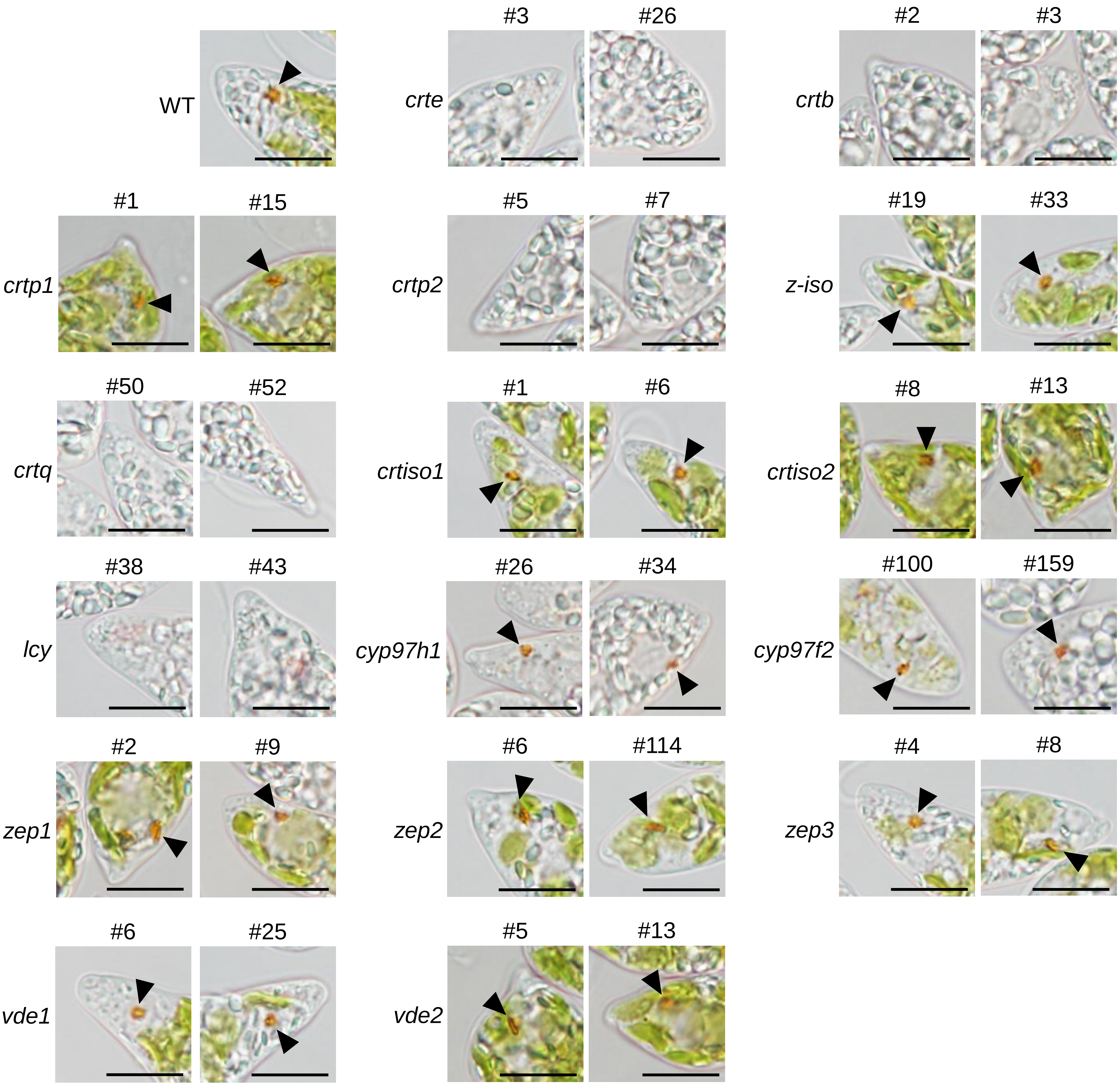

Supplemental Figure S3. Images of the eyespots of each knockout mutant. Images were generated by magnifying eyespot-containing areas shown in Figure 2. Wild-type (WT) and mutant cells were grown in KH medium under continuous light (40 µmol photons m^{-2} s^{-1}) at 26˚C for 10 days. Arrowheads indicate the eyespot apparatus. Scale bars, 10 µm.

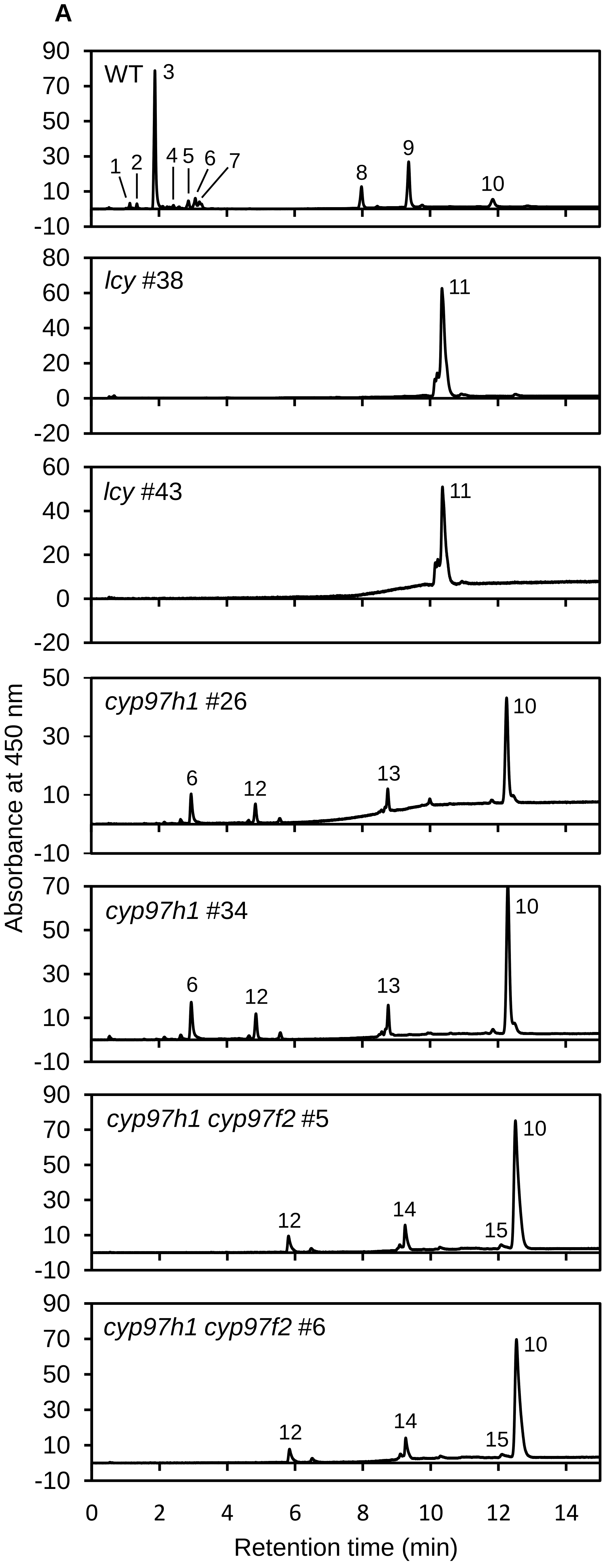

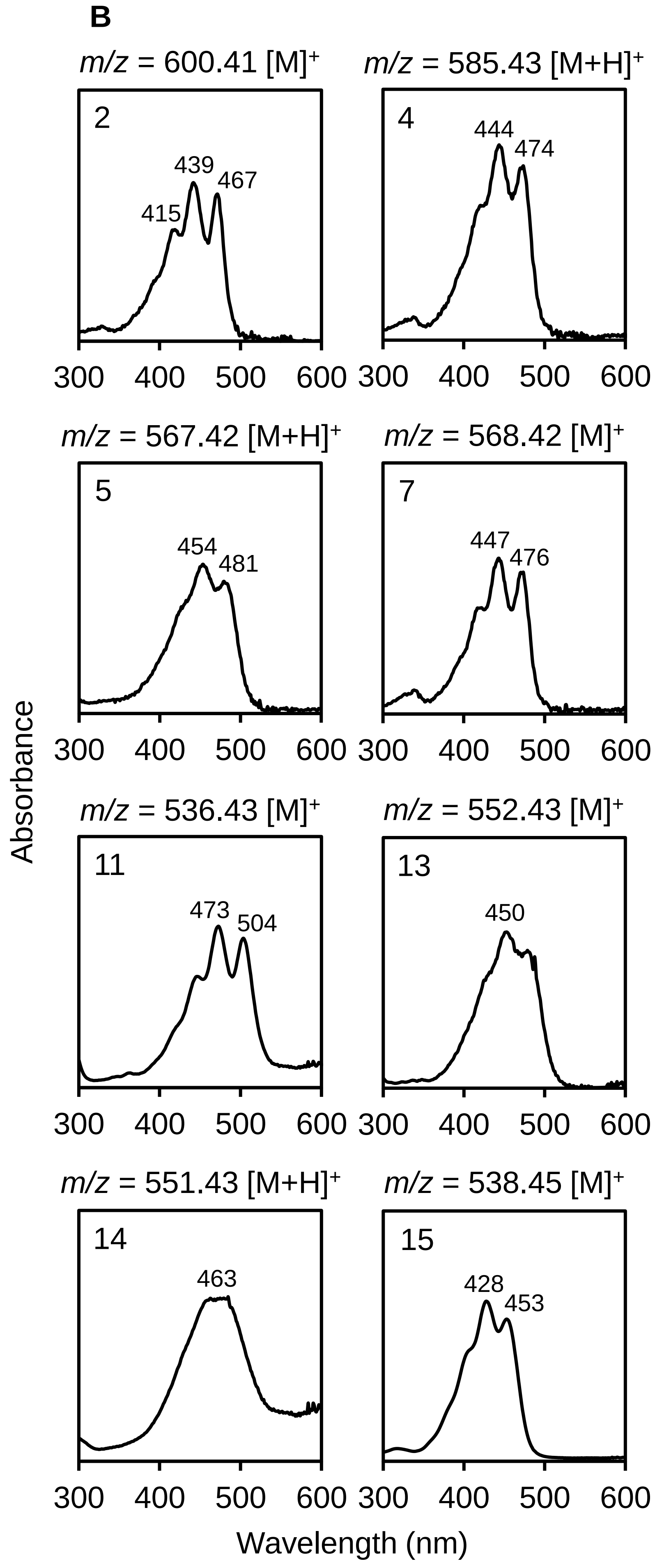

Supplemental Figure S4. LC/MS analysis of carotenoids extracted from the wild type (WT) and each mutant. Cells were grown in KH medium under continuous light (40 µmol photons m^{-2} s^{-1}) at 26˚C for 10 days. (A) HPLC chromatograms of the carotenoid extracts at 450 nm absorbance. (B) UV-visible spectra and *m/z* values from the MS spectra of individual carotenoids (peaks 2, 4, 5, 7, 11, 13–15). Spectral data of other carotenoids were identical to previous results (Tamaki et al., 2020). 1, Neoxanthin; 2, violaxanthin; 3, diadinoxanthin; 4, antheraxanthin; 5, diatoxanthin; 6, zeaxanthin; 7, lutein; 8, chlorophyll *b*; 9, chlorophyll *a*; 10, β-carotene; 11, lycopene; 12, canthaxanthin; 13, β-cryptoxanthin; 14, echinenone; and 15, β-zeacarotene.

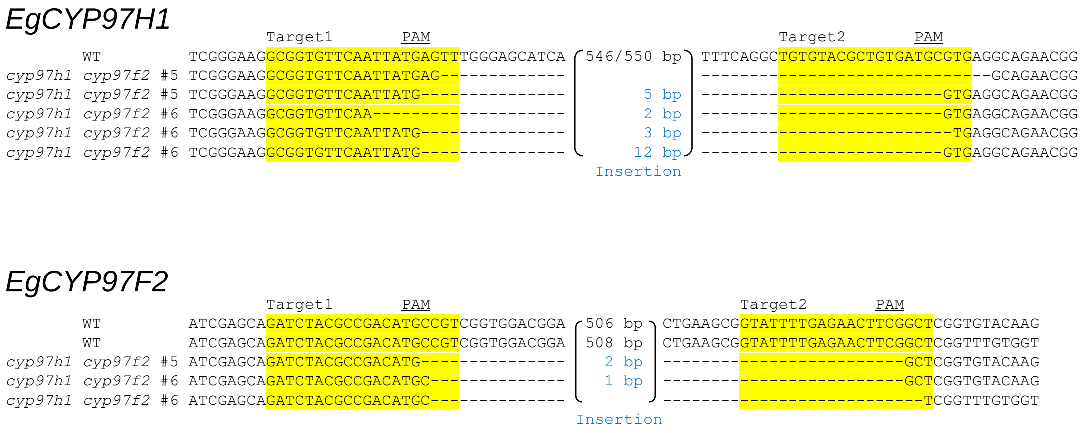

Supplemental Figure S5. The nucleotide sequence alignment of PCR fragments amplified from the wild type (WT) and *cyp97h1 cyp97f2* double mutants. The guide RNA (gRNA) target sequences are highlighted in yellow. Blue letters represent the length of the insertion sequence. PAM, protospacer adjacent motif.

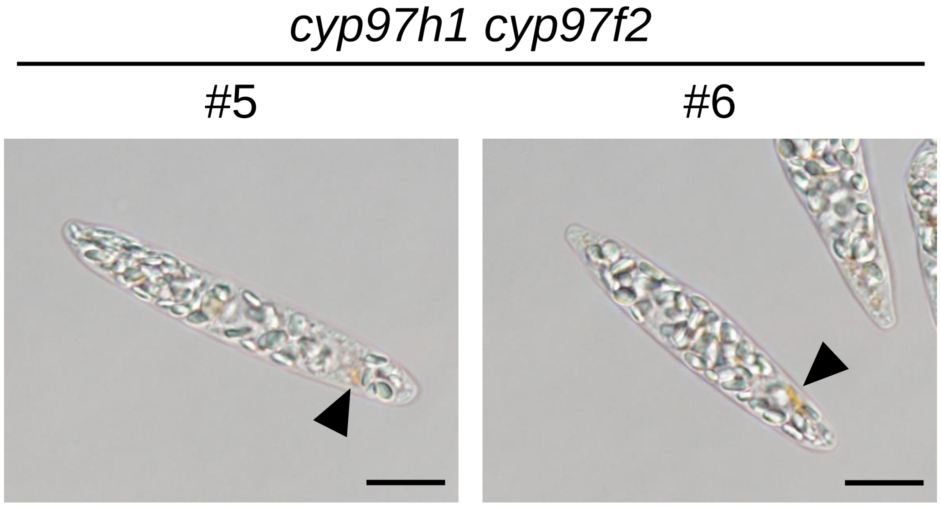

Supplemental Figure S6. Rarely observed red eyespots of *cyp97h1 cyp97f2* double mutants. Cells were grown in KH medium containing 0.1% (v/v) ethanol under continuous light (40 μmol photons m^{-2} s^{-1}) at 26˚C for 10 days. Arrowheads indicate the eyespot apparatus. Scale bars, 10 μm.

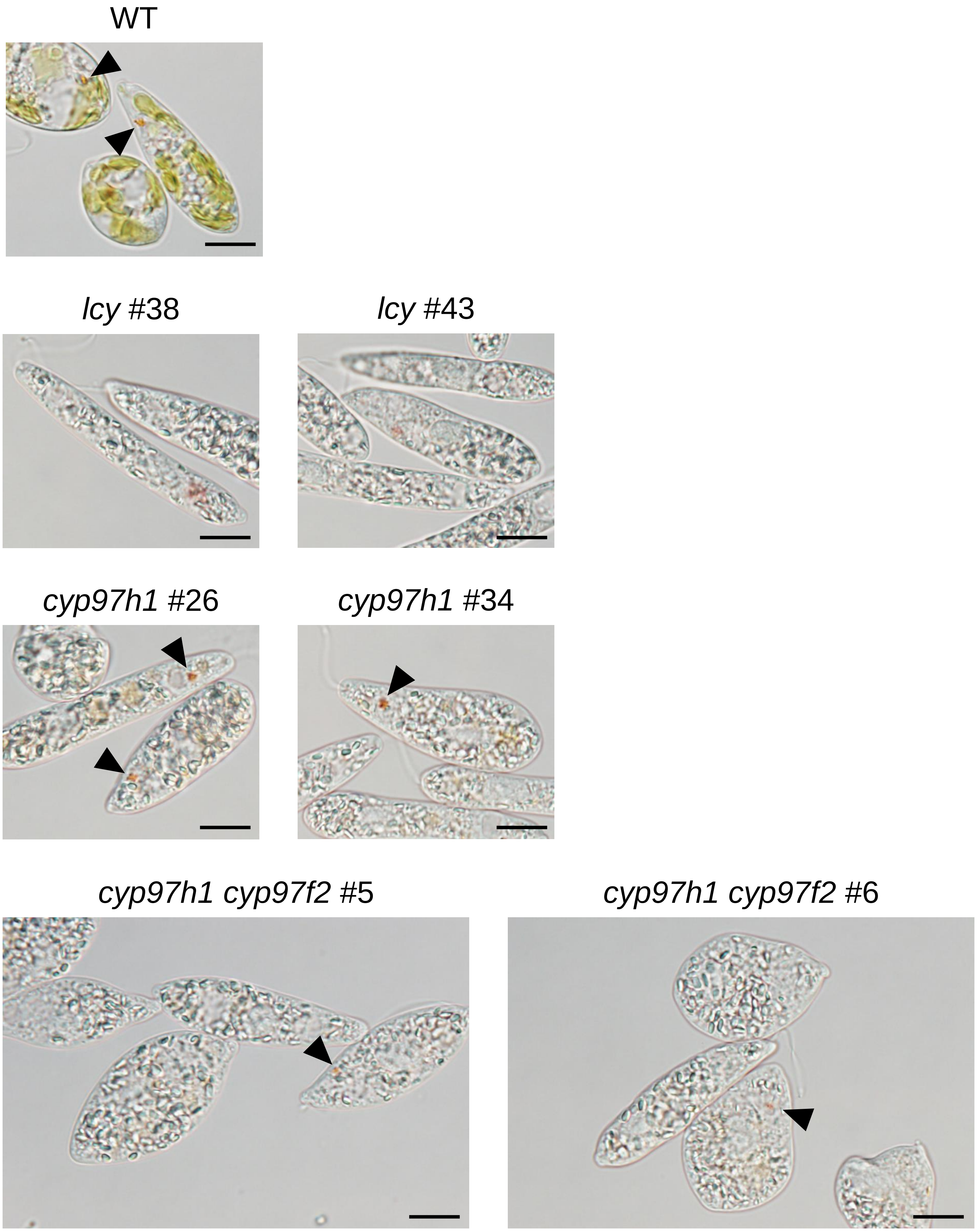

Supplemental Figure S7. Cell appearances of carotenoid biosynthetic gene knockout mutants. Wild-type (WT) and mutant cells were grown in modified CM medium containing 0.1% (v/v) ethanol under continuous light (40 µmol photons m^{-2} s^{-1}) at 26˚C for 10 days. Arrowheads indicate the eyespot apparatus. Scale bars, 10 µm.

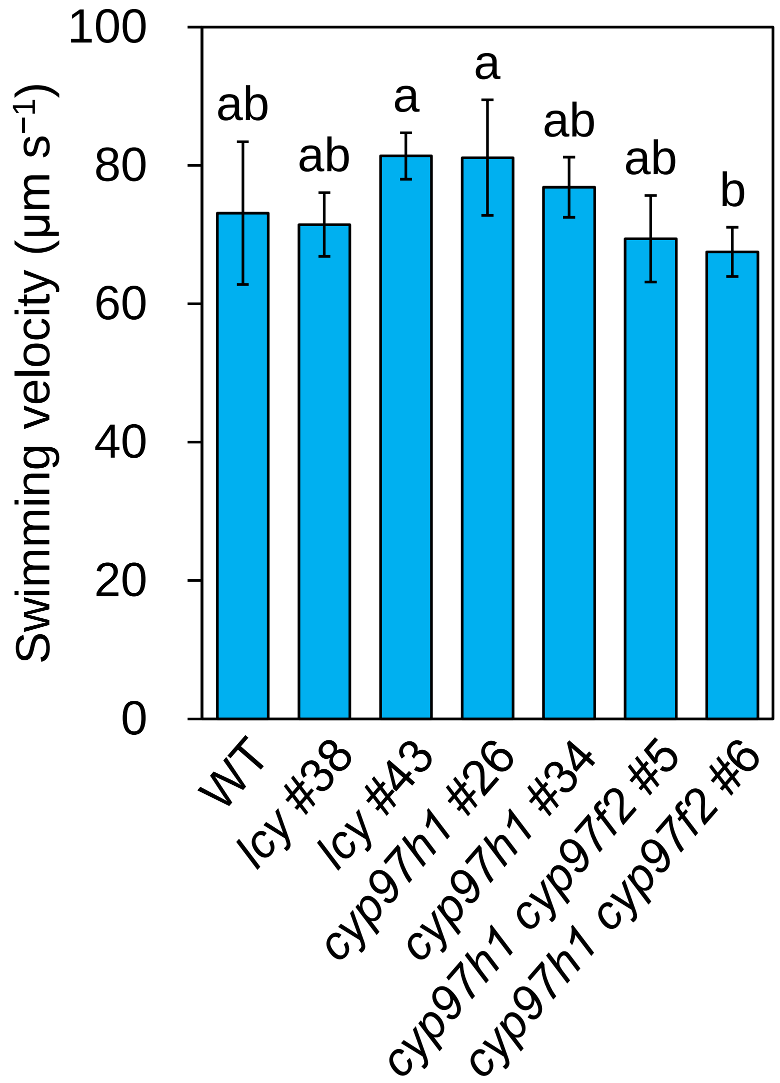

Supplemental Figure S8. The swimming velocities of carotenoid biosynthetic gene knockout mutants during phototactic analysis. Swimming velocity was quantified as described previously (Kato et al., 2020). Values are presented as the mean ± SD (*n*=5). Values with different letters are significantly different from other strains according to the Tukey-Kramer multiple comparison test ($p < 0.05$).

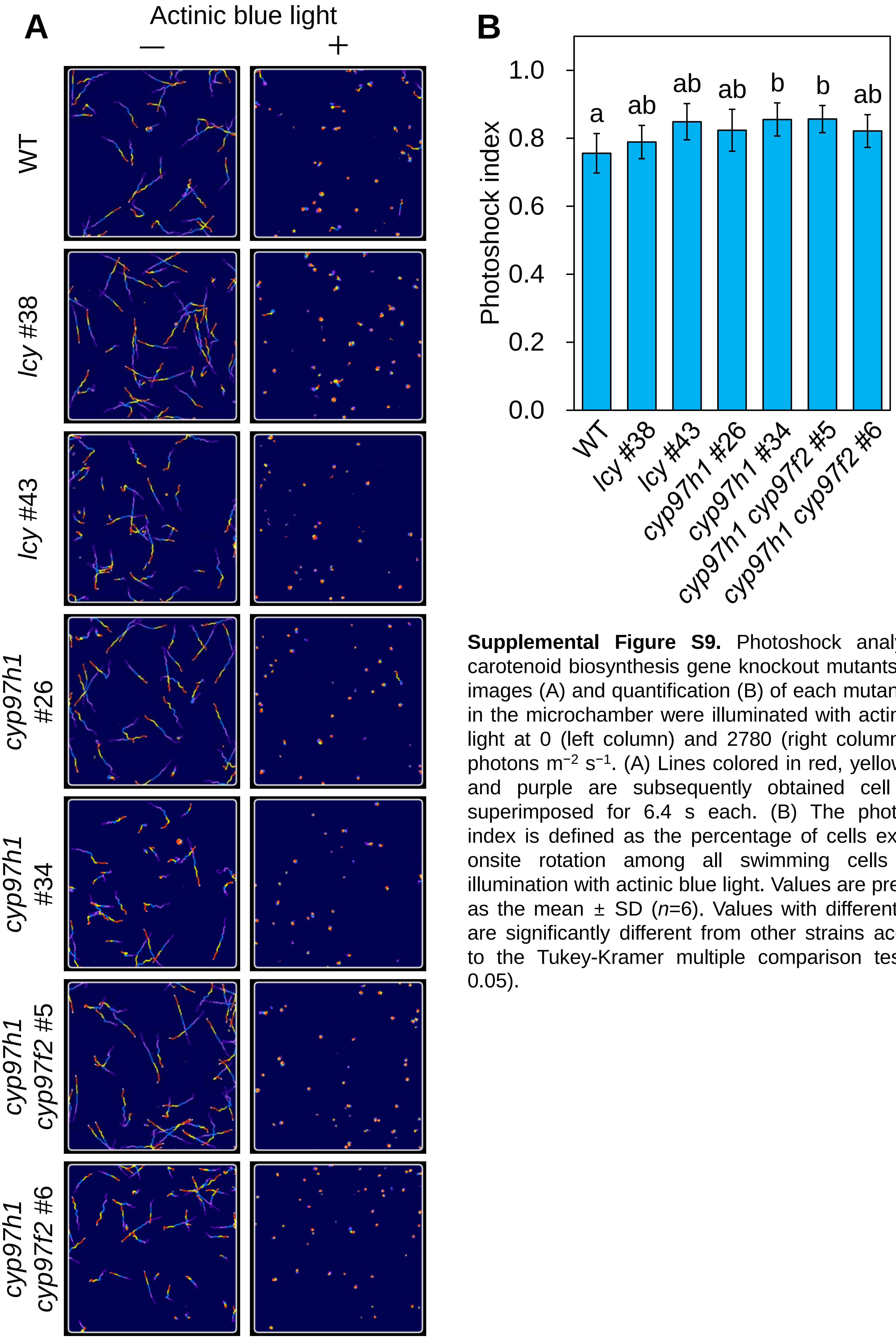

Supplemental Figure S9. Photoshock analysis of carotenoid biosynthesis gene knockout mutants. Trace images (A) and quantification (B) of each mutant. Cells in the microchamber were illuminated with actinic blue light at 0 (left column) and 2780 (right column) µmol photons m^{-2} s^{-1}. (A) Lines colored in red, yellow, blue, and purple are subsequently obtained cell traces superimposed for 6.4 s each. (B) The photoshock index is defined as the percentage of cells exhibiting onsite rotation among all swimming cells during illumination with actinic blue light. Values are presented as the mean ± SD (n=6). Values with different letters are significantly different from other strains according to the Tukey-Kramer multiple comparison test (p < 0.05).

Supplemental Table S1. List of putative carotenoid biosynthetic genes knocked out in *E. gracilis*.

Gene	Accession number	Function/Annotation	E-value [Uniprot ID of query]	Reference
EgcrtE	LC700283<br>LC700284<br>LC700285	geranylgeranyl pyrophosphate synthase	—	Kato et al., 2016
EgcrtB	LC700286<br>LC700287	phytoene synthase	—	Kato et al., 2016
EgcrtP1	LC700288	phytoene desaturase	—	Kato et al., 2019
EgcrtP2	LC700289<br>LC700290	phytoene desaturase	—	Kato et al., 2019
EgZ-ISO	LC700291<br>LC700292<br>LC700293	ζ-carotene isomerase	—	Sugiyama et al., 2019
EgcrtQ	LC700294<br>LC700295	ζ-carotene desaturase	—	Kato et al., 2019
EgcrtISO1	LC700296<br>LC700297	prolycopene isomerase	3e-60<br>[CRTSO_SOLLC]	—
EgcrtISO2	LC700298<br>LC700299	prolycopene isomerase	2e-43<br>[CRTSO_SOLLC]	—
EgLCY	LC700300	lycopene cyclase	—	Tamaki et al., 2021b
EgCYP97H1	LC700301<br>LC700302	β-carotene hydroxylase	—	Tamaki et al., 2019
EgCYP97F2	LC700303<br>LC700304	β-carotene hydroxylase	1e-105 [LUT5 _ARATH]	—
EgZEP1	LC700305	zeaxanthin epoxidase	5e-68	—

		(diatoxanthin epoxidase)	[ZEP_ARATH]	
EgZEP2	LC700306	zeaxanthin epoxidase (diatoxanthin epoxidase)	5e-11 [ABA2_PRUAR]	—
EgZEP3	LC700307 LC700308	zeaxanthin epoxidase (diatoxanthin epoxidase)	7e-16 [A0A2V0P950_9CHLO]	—
EgVDE1	LC700309	violaxanthin de-epoxidase (diadinoxanthin de-epoxidase)	1e-47 [VDE_ARATH]	—
EgVDE2	LC700310 LC700311	violaxanthin de-epoxidase (diadinoxanthin de-epoxidase)	2e-84 [I1K4S1_SOYBN]	—

Supplemental Table S2. Primer sequences used in this study.

Primer	Sequence
Primer a	5'-CTAATACGACTCACTATAG-(20 bp target sequence)-GTTTTAGAGCTAGAAATAGCAAGTTAAAATA-3'
Primer b	5'-AAAGCACCGACTCGGTGCCACTTTTTCAAGTTGATAACGGACTAGCCTTATTTTAACTTGCTATTTCTA-3'
Primer c	5'-AAAAGCACCGACTCGGTGCC-3'
EgcrtE-F	5'-AATACCCTTCTGTCTCATCCGAC-3'
EgcrtE-R	5'-CAAGTGCATCTCACAAACACTGAT-3'
EgcrtB-F	5'-AGACGAAGACCTTCAATGCACAG-3'
EgcrtB-R	5'-AAGCAATGCTATGGCTGGATCTT-3'
EgcrtP1-F	5'-AAGCCAGAACAAATAGATCAACCAC-3'
EgcrtP1-R	5'-TACCTGGAAGAGGTTCATCATGTT-3'
EgcrtP2-F	5'-CAATTGCATAATGGAACCTCTTCT-3'
EgcrtP2-R	5'-CCATGGCCAAATCTTCTTGTG-3'
EgZ-ISO-F	5'-GACATCCACCACTCCATATGTTG-3'
EgZ-ISO-R	5'-TTCTATTCGGTGTGCTGCAAACT-3'
EgcrtQ-F	5'-TTCAACATTGGTGTGTTTTGTGC-3'
EgcrtQ-R	5'-GTTCAATCATCCTCACAACAGTGA-3'
EgcrtISO1-F	5'-TGTTATATTTTGGGAGTTGTCTCGC-3'
EgcrtISO1-R	5'-GCAAACATTGGAACACATTTGC-3'
EgcrtISO2-F	5'-AAGAAATTTCCTGCTCGTGATAC-3'
EgcrtISO2-R	5'-CTTCACATCCACTTTCATTCACAC-3'
EgLCY-F	5'-TTTCCATGTTGTATAATCAGCACTT-3'
EgLCY-R	5'-ATCCATAGTCATCCGTAGTTTCAGG-3'
EgCYP97H1-F	5'-TTTATCCAGGTACAGCACAGTCG-3'
EgCYP97H1-R	5'-TTACAGAGAAATTTGGCTTGCTG-3'
EgCYP97F2-F	5'-GTCACATAACACTGCTGCTGTTT-3'
EgCYP97F2-R	5'-ATTGGAGCCAAAACATATGCAC-3'
EgZEP1-F	5'-CTGGGAGTTCAGGTACCGGTAAT-3'
EgZEP1-R	5'-TTATCCCAACTGCAATTTTAGGCA-3'
EgZEP2-F	5'-ACACCAGCACGTTGAACAGAATA-3'
EgZEP2-R	5'-ATTCCTGTGTATCCTGTGAAGTCC-3'
EgZEP3-F	5'-TCGTGTAGAAACTGTCCCAAAATG-3'
EgZEP3-R	5'-AACTATGGCAATGGATCTTTGGTC-3'
EgVDE1-F	5'-CACTCTCACTCAAATACATGTGGC-3'
EgVDE1-R	5'-AAACGTGAGATTCGGGATGTTG-3'

EgVDE2-F	5'-ATGGTGAAGAATAACCAGCTGGAT-3'
EgVDE2-R	5'-CTATGCTTCGAGTGGTGTTCAAAT-3'

Supplemental Table S3. Target sequences for gRNA synthesis.

Gene	Sequence
EgcrtE-1	5'-ACCCCGTGGCATCCGCCTCC-3'
EgcrtE-2	5'-CCTCTCCACAGGAGGCGTTG-3'
EgcrtE-3	5'-ACGCTTGTTGCAAATGAGCC-3'
EgcrtB-1	5'-TAATGAGGTCGAGAAGATTA-3'
EgcrtB-2	5'-CCGTCGCACGGATGAGATTG-3'
EgcrtP1-1	5'-GGATACCGACAACTTCCGCG-3'
EgcrtP1-2	5'-GCATCTCCCCATTGTCCTCG-3'
EgcrtP2-1	5'-CAACCATTTGCATCTCACCC-3'
EgcrtP2-2	5'-GCAGCCAAGTTCTCGTCAGG-3'
EgZ-ISO-1	5'-GCTGCCCATTACCTCTTTAT-3'
EgZ-ISO-2	5'-GCAAGTTCGCCACGGGTCGA-3'
EgZ-ISO-3	5'-GCAAGTTCGCCATGGGTCGA-3'
EgcrtQ-1	5'-GGAAAGCACCTCCGTCGACC-3'
EgcrtQ-2	5'-GCTGGCGGCGGACGGCAAGA-3'
EgcrtISO1-1	5'-ATCATAGACGCGCTGGTCCG-3'
EgcrtISO1-2	5'-GCGACGGTGTGGGACACGTT-3'
EgcrtISO2-1	5'-CGCGGCCTTCAGCGGTGCGC-3'
EgcrtISO2-2	5'-CGTGACAGCACCGTTCGCGG-3'
EgLCY-1	5'-AGGCCACGCTGGGCATCCGC-3'
EgLCY-2	5'-GAGGCGACCCGACTTCCGCG-3'
EgCYP97H1-1	5'-GCGGTGTTCAATTATGAGTT-3'
EgCYP97H1-2	5'-TGTGTACGCTGTGATGCGTG-3'
EgCYP97F2-1	5'-GATCTACGCCGACATGCCGT-3'
EgCYP97F2-2	5'-GTATTTTGAGAACTTCGGCT-3'
EgZEP1-1	5'-AATGATGCCCAACCTCGGCC-3'
EgZEP1-2	5'-GGCTGCTTCAGGAGTACTAC-3'
EgZEP2-1	5'-GGGTATCGTGTGCCGCTTCG-3'
EgZEP2-2	5'-CAGCGGAGATCAACGCGGAC-3'
EgZEP3-1	5'-GGCATGACAATCCTGACCCT-3'
EgZEP3-2	5'-CGCCCACAACGTCCGCATCA-3'
EgVDE1-1	5'-GCCACACGTTTACGGTGGAC-3'
EgVDE1-2	5'-CGTCGACCAGTCGTGGGTCC-3'
EgVDE2-1	5'-GGTGGCCTGTATGCAGTCCC-3'
EgVDE2-2	5'-CATCACCTCGTATGAGAATG-3'

Three types of gRNA for *EgcrtE* were used as described in Figure 3. *EgZ-ISO-2* and *EgZ-ISO-3* gRNAs include a 1-bp substitution, and thus *E. gracilis* was treated with half the amount (2 µg each) of *EgZ-ISO-2* and *EgZ-ISO-3* gRNAs to knock out *EgZ-ISO*.
