# Supplementary material for: Zeaxanthin is required for eyespot formation and phototaxis in Euglena gracilis
Source: Plant Physiol. 2023 Jan 6;191(4):2414–26. doi: 10.1093/plphys/kiad001 (PMC10069888; doi:10.1093/plphys/kiad001)
Supplement: kiad001_Supplementary_Data [file kiad001_supplementary_data.zip › Supplemental Movie Legends.pdf]

Supplemental Movie S1. Movement tracks of wild-type (WT) *E. gracilis* illuminated with blue light at 0 or 360 μmol photons m^{-2} s^{-1}.

Supplemental Movie S2. Movement tracks of *E. gracilis cyp97h1* mutants illuminated with blue light at 0 or 360 μmol photons m^{-2} s^{-1}.

Supplemental Movie S3. Movement tracks of *E. gracilis lcy* mutants illuminated with blue light at 0 or 360 μmol photons m^{-2} s^{-1}.

Supplemental Movie S4. Movement tracks of *E. gracilis cyp97h1 cyp97f2* double mutants illuminated with blue light at 0 or 360 μmol photons m^{-2} s^{-1}.
